# Genetic, developmental, and neural changes underlying the evolution of butterfly mate preference

**DOI:** 10.1371/journal.pbio.3002989

**Published:** 2025-03-11

**Authors:** Nicholas W. VanKuren, Nathan P. Buerkle, Wei Lu, Erica L. Westerman, Alexandria K. Im, Darli Massardo, Laura Southcott, Stephanie E. Palmer, Marcus R. Kronforst

**Affiliations:** 1 Department of Ecology & Evolution, The University of Chicago, Chicago Illinois, United States of America; 2 Department of Organismal Biology & Anatomy, The University of Chicago, Chicago, Illinois, United States of America; 3 Department of Physics, The University of Chicago, Chicago, Illinois, United States of America; New York University, UNITED STATES OF AMERICA

## Abstract

Many studies have linked genetic variation to behavior, but few connect to the intervening neural circuits that underlie the arc from sensation to action. Here, we used a combination of genome-wide association (GWA), developmental gene expression, and photoreceptor electrophysiology to investigate the architecture of mate choice behavior in *Heliconius cydno* butterflies, a clade where males identify preferred mates based on wing color patterns. We first found that the GWA variants most strongly associated with male mate choice were tightly linked to the gene controlling wing color in the *K* locus, consistent with previous mapping efforts. RNA-seq across developmental time points then showed that seven genes near the top GWA peaks were differentially expressed in the eyes, optic lobes, or central brain of white and yellow *H. cydno* males, many of which have known functions in the development and maintenance of synaptic connections. In the visual system of these butterflies, we identified a striking physiological difference between yellow and white males that could provide an evolutionarily labile circuit motif in the eye to rapidly switch behavioral preference. Using single-cell electrophysiology recordings, we found that some ultraviolet (UV)-sensitive photoreceptors receive inhibition from long-wavelength photoreceptors in the male eye. Surprisingly, the proportion of inhibited UV photoreceptors was strongly correlated with male wing color, suggesting a difference in the early stages of visual processing that could plausibly influence courtship decisions. We discuss potential links between candidate genes and this physiological signature, and suggest future avenues for experimental work. Taken together, our results support the idea that alterations to the evolutionarily labile peripheral nervous system, driven by genetic and gene expression differences, can significantly and rapidly alter essential behaviors.

## Abstract 2

Few studies that link genetic variation to behavior also dissect the neural circuits that underlie the arc from sensation to action. This study of the architecture of mate choice behavior in *Heliconius cydno* butterflies suggests that alterations to the peripheral nervous system, driven by genetic and gene expression differences, can alter essential behaviors.

## Introduction

Behavioral evolution requires genetic variation that ultimately alters the neural circuits mechanistically responsible for generating differences in behavior. Many studies have mapped the genetic basis for behavioral evolution [1–4] or associated differences in neural physiology with divergent behavior [5–7], but the links between each of these layers of variation are often missing. The peripheral nervous system appears to be an especially labile target for evolutionary modification [8–11]. Shifts in receptor sensitivity can avoid the potentially deleterious effects associated with changing complex brain circuits while still enabling changes in the perception and distinguishability of sensory stimuli. However, simple shifts in receptor sensitivity may be insufficient to enact large behavioral changes, instead requiring more significant changes in how downstream circuits process sensory information [5,6,12,13]. Understanding how these more complex changes emerge requires an integrative approach combining genetics, transcriptomics, and neurobiology that can reveal how genetic variation affects the functional organization of the brain.

The co-evolution of wing color and mate choice in Neotropical *Heliconius* butterflies presents an excellent system to integrate multiple approaches to studying behavioral evolution. *Heliconius* butterflies evolved myriad bold wing color patterns that warn predators of their toxicity and mediate mimicry, but also serve as the primary signals used for mate choice [14–19]. Visual perception of wing color patterns is the critical first step in mate choice, as males preferentially court females with the same wing color [14,15,20]. The genes controlling most color pattern variation have been identified and extensively studied, but only recent mapping studies have begun to identify the genetic loci associated with mate choice variation [15,18,19]. These studies provided two important observations. First, loci associated with mate choice variation are often tightly linked to the loci that control color patterns used for mate choice [15,16,19–22]. Second, Rossi and colleagues [19] showed that genetic and expression variation of *regucalcin1*, a gene expressed throughout the nervous system, was associated with species-specific mate choice behavior in *Heliconius melpomene*, *Heliconius cydno*, and *Heliconius timareta*. Together, these studies suggest where to look for putative mate choice genes in the genome and visual circuits. However, the direct links between genetic variation, expression variation, development, and behavior remain elusive.

**Fig 1:**
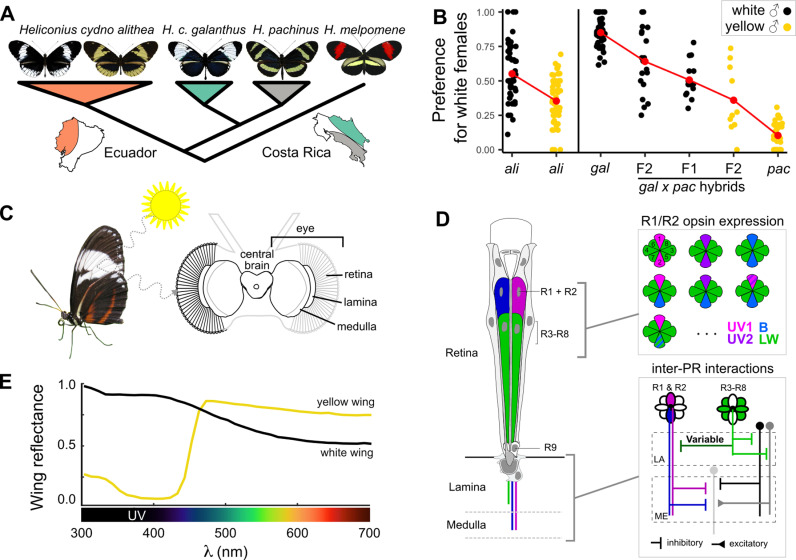
Color pattern and visual mate choice behavior in the *Heliconius cydno* clade. (**A**) The *cydno* complex and its sister taxon, *Heliconius melpomene*. (**B**) Male preference, i.e., the proportion of courts directed at white females [15,16]. Each point represents preference of one male; red points are means. (**C**) Visual perception of wing color and organization of the *Heliconius* visual system. Light received through hundreds of individual ommatidia in the retina is transmitted through multiple layers of the optic lobe to the central brain. (**D**) The eye is organized into ommatidia containing nine photoreceptors (left), with distinct ommatidial types typically defined by the opsin expression patterns in the R1 and R2 cells (right). R1/R2 axons bypass the lamina (LA) and project directly to the medulla (ME), while R3-8 project only to the lamina where they can also make inter-photoreceptor synaptic connections with R1/R2 axons [23,24,25]. (**E**) Spectral reflectance of white and yellow *H. cydno* wings primarily differ in the UV region. Raw data and code used to generate panels A and E can be found in Dryad repository dryad.z8w9ghxjz.

Here, we investigated the genetic and neurobiological basis of mate choice variation within the *H. cydno* clade of butterflies (Fig 1). *Heliconius pachinus* and *Heliconius cydno galanthus* are yellow- and white-winged sister species in which males have strong preference for females with conspecific wing colors. Previous mapping experiments showed that both color and mate choice variation are most strongly associated with the *K* locus, a narrow region on chromosome 1 [15]. In contrast, *Heliconius cydno alithea* is polymorphic for yellow and white wings and males display variable mate choice behavior: while yellow *H. c. alithea* males have strong preference for yellow females, white *H. c. alithea* males court yellow and white females equally (Fig 1B). This latter result is consistent with the fact that most white *alithea* are heterozygous at the *K* locus, and mirrors both the behavior and genetics of *galanthus*/*pachinus* F1 hybrids [15,16]. We previously showed that *aristaless-1*, a gene located in the *K* locus, controls the switch between recessive yellow and dominant white wing colors [26,27]. However, the gene(s) controlling mate choice variation remained unknown.

Mate choice behavior thus appears to be associated with one or a few major-effect loci, but how this genetic variation manifests as differences in the underlying neural circuitry remains unknown. Butterfly visual systems vary extensively across species both in eye organization [28,29] and in the size of the brain [30], highlighting that causal differences could occur anywhere along the visual processing pathway (Fig 1C). Differences in eye organization are especially well characterized within *Papilio* and *Heliconius*, with variation in screening pigments [31–33], opsin expression [34,35], and co-expression of multiple opsins within a single photoreceptor (PR) [36,37] contributing to at least six distinct ommatidial types in *Heliconius* alone (Fig 1D) [29,38,39]. In *Heliconius*, these opsins include a long wavelength sensitive opsin (LW), a blue sensitive opsin, and paralogous UV sensitive opsins (UV1 and UV2) tuned to ~ 355 nm and ~ 390 nm, respectively [28,40]. Additionally, physiological and anatomical data from other butterfly species have revealed inhibitory inter-PR synaptic connections between cells with different spectral sensitivities [23–25,41–43]. These interactions between PRs generate color-opponent-like receptive fields, although the impact on downstream visual computations and behavior remains unexplored.

Here, we took an integrative approach to attempt to identify the causes of *H. cydno* mate choice variation. Using a combination of genome-wide association (GWA), developmental transcriptomics, and PR electrophysiology, we sought to comprehensively characterize variation in the *Heliconius* visual system. We found genetic and gene expression variation associated with mate choice behavior on at least four chromosomes, with the largest effect genes in the *K* locus. Furthermore, we identified striking differences in inter-PR inhibition between *H. cydno* males that were strongly correlated with their mate preference. Overall, our results suggest that one outcome of *K* locus variation is differences in inter-PR inhibition of UV PRs that contribute to variable mate choice behavior, and provide important hypotheses about the links between genetic variation, peripheral visual system variation, and a critical visual behavior.

## Results

### *Heliconius cydno alithea* mate choice variation is strongly associated with the *K* locus

**Fig 2:**
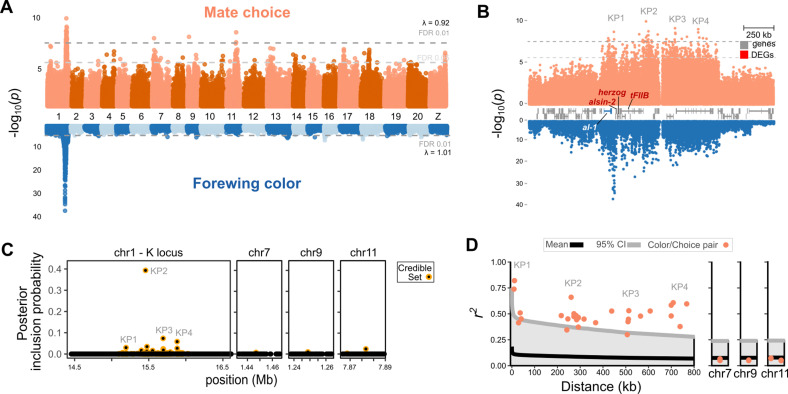
Genetic architecture of *Heliconius cydno alithea* male mate choice variation. (**A**, **B**) Genome-wide association (GWA) for *H. c. alithea* mate choice and forewing color genome-wide (**A**) and at the *K* locus (**B**) using 1,529 courtship events from 57 yellow and 56 white males. Chromosome numbers are shown between the plots. Lambda values (*λ*) are genomic inflation factors for each analysis, indicating no *p-*value inflation in the dataset despite potential residual population stratification (S1 Fig). *K* locus peak (KP) labels referenced in the text are shown above the plot. DEGs: differentially expressed genes—see Fig 3. KP2 was 25 kb downstream of the ortholog of *Drosophila melanogaster Hasp*; KP3 was 15 kb upstream of the transcription factor *senseless-2* and *RNA pseudouridylate synthase domain containing protein 2* (*rpusd2*); and KP4 was within an *rpusd2* intron. FDR: Benjamini–Hochberg false discovery rate cutoff. (**C**) Posterior inclusion probability for each variant in the chromosome 1, 7, 9, and 11 peaks, calculated using *SuSiE-RSS* [44]. PIP values indicate the probability that each variant is predictive of the input phenotypes (choices in this case). Higher PIP values indicate higher predictive value. We calculated PIP values for top variants in the *K* locus as well as the peaks on chromosomes 7, 9, and 11. (**D**) Pairwise linkage disequilibrium between top color and preference variants relative to the empirical LD in the *K* locus at each distance. We only show values between the top color variant and each of the 34 choice variants at FDR <  0.01. Patterns were similar between the second and third ranked color variants and are not shown. *K* locus variants are shown in the left panel while the chromosome 7, 9, and 11 variants are shown in subsequent panels relative to empirical unlinked marker LD. Raw data, code, and further exploration of the data can be found in S1 and S2 Tables, S1 and S2 Figs, and Dryad repository dryad.z8w9ghxjz.

Previous quantitative trait locus mapping in *H. c. galanthus* and *H. pachinus* showed that both wing color and male preference variation were linked to the ~ 2 Mb *K* locus on chromosome 1 [15]. Although color and preference were also strongly correlated in *H. c. alithea*, the genetic architecture of preference variation in this subspecies, and whether it mirrored the situation *H. c. galanthus* and *H. pachinus*, remained unknown [15,16]. We began mapping color and preference variation in *H. c. alithea* by performing a GWA for color and male mate choice using 1,529 courtship events from 57 yellow and 56 white males (Figs 2 and S1 and S2; S1 Table) [16]*.* These males were tested for their preference by Chamberlain and colleagues (2009), then re-sequenced for this study.

Consistent with our previous study, *H. c. alithea* forewing color variation was strongly associated with a single narrow region within the *K* locus, 21 kb downstream of *al-1* (Fig 2) [26]. In contrast, *H. c. alithea* mate choice variation was associated with genetic variation on four chromosomes at a false discovery rate (FDR) of 1% (Fig 2) [15,46]. The most significant associations were found in the *K* locus, while secondary peaks were found on chromosomes 7, 9, and 11. Within the *K* locus, significant variants localized to four discrete *K* locus peaks (KP): KP1 was near, but not coincident with, the top color variants while KP2, KP3, and KP4 were 240 kb, 520 kb, and 790 kb away, respectively (Fig 2B).

In addition to the *K* locus peak, mate choice was also strongly associated with variation on chromosomes 7, 9, and 11 (Figs 2 and S2). Top variants on chr7 fall within an intron of *diacyl glycerol kinase*, a gene encoding an enzyme involved in cell membrane homeostasis and intracellular signaling. The chr11 variants span 350 kb and 19 genes, with the top variants falling within introns of a potential acyl transferase (*CG17707*) and *collagen 11A1* and 2.5 kb downstream of *ubiquitin conjugating enzyme 2M* (*UBE2M;* S2 Fig). Although the chr7 and chr11 genes had no immediately obvious links to vision or courtship behavior, we were intrigued to find that the top variant on chr9 falls within an intron of the transcription factor *spineless*, which controls stochastic choice of opsin expression in R1/R2 cells in *Papilio* swallowtail butterfly eyes (Fig 1) [47]. Thus, plausible mate choice genes may also be found outside of the major effect gene(s) within the *K* locus.

Consistent with the *K* locus having the main effect on mate choice variation, white alleles at the top KP2 variant had the highest marginal effects on male mate choice (0.11, 95%CI 0.05–0.18) followed by 0.08 (0.04–0.12), 0.07 (0.03–0.11), and 0.02 (−0.02 to 0.06) at the top chr9, chr11, and chr7 variants, respectively. Although these estimates may be somewhat inflated due to the Beavis effect [48], they suggest that each white allele in KP2 reduced the probability of choosing white females by ~ 11%. These results were altogether consistent with previous mapping studies linking mate choice to the *K* locus in *H. c. galanthus* and *H. pachinus*, but the increased resolution from this population-level analysis strongly suggested that separate, discrete genetic loci control color and behavior variation.

### Color and choice are associated with physically separate, but genetically linked variants

To better estimate which of the significant GWA variants was potentially causal for mate choice variation, we performed fine-mapping of the *K* locus, chr7, chr9, and chr11 variants using *SuSiE-RSS* [49]. This approach calculates the likelihood of each variant contributing to mate choice variation given the GWA coefficient estimates and linkage disequilibrium (LD) between those variants, outputting statistically credible sets of putative causal sites. *SuSiE-RSS* identified 160 variants in the final credible set, out of 61,824 analyzed. These results largely mirrored the GWA, but strongly suggested that the top KP2 variant, a C/T single-nucleotide polymorphism (SNP) 240 kb away from the top color variant, was most predictive of male mate choice (Fig 2C) [44].

The top mate choice and color variants were not coincident, suggesting that physically separate loci control these two traits. Yet, theory predicts that the correlation between loci controlling mate preference and cue will rapidly decay in the absence of mechanisms that maintain LD between them [50]. Despite the top color and choice *K* locus variants being up to 790 kb apart, most pairs of color and choice variants were in extremely high LD relative to empirical levels of LD in the *K* locus (Fig 2D). LD between the top color variant and KP4 variants, for example, was over 10 times higher than the *K* locus average of 0.06 for similarly spaced variants (Fig 2D). LD across the *K* locus, measured in sliding windows of *r*^*2*^ or *D*′, was not generally increased relative to the genome-wide average; however, and we found no evidence for inversions or other genomic features that could easily explain this high pairwise LD (Figs 2D and S3 and S4). Together, these results suggested that the genomic basis for genetic coupling in *H. cydno* comprises physically separate, but genetically linked variants that control wing color and male color preference.

### Differential expression of genes near top choice variants

**Fig 3:**
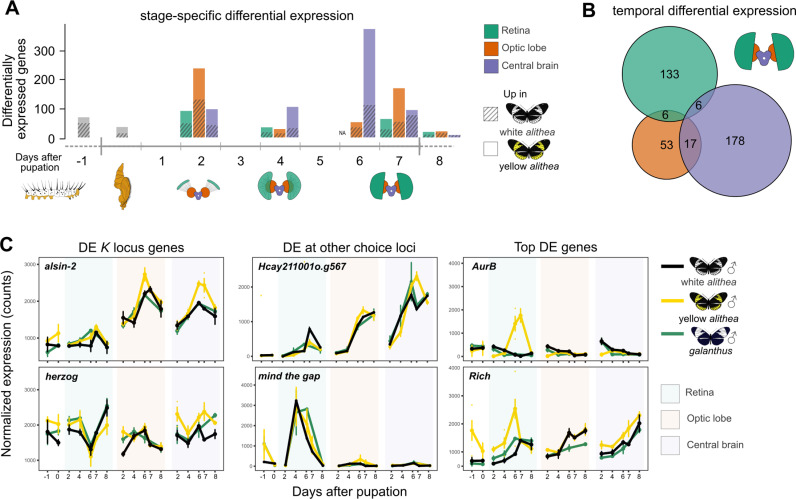
Differential gene expression between yellow and white *Heliconius cydno alithea* males. (**A**) Stage- and tissue-specific differential expression between yellow and white *H. c. alithea* males identified using DESeq2 and a global FDR cutoff of 0.05. (**B**) Genes with significantly different temporal expression patterns in yellow and white *H. c. alithea* males in each tissue, calculated using maSigPro. Overlap represents genes with significantly different expression profiles in all overlapping tissues, represented by an Euler diagram. maSigPro fits a curve to each gene in each group (i.e., yellow males, yellow females, white male, white females) over all developmental stages. A gene is considered DE if one or more of its regression coefficients is significantly different between yellow and white *H. c. alithea* males. (**C**) Expression profiles of DE *K* locus genes (left), other top choice locus genes (middle), and additional top DE candidate genes (right) (see Figs 2 and S5). *AurB* is a protein kinase expressed throughout the fly nervous system [45]; *Rich* is a Rab guanine nucleotide exchange factor required for synapse formation and function from inner photoreceptor cells (fly R7 +  R8, butterfly R1/2 +  R9) to medulla neurons [41]. The full list of DE genes can be found in S4–S7 Tables.

All significant choice variants were intergenic or intronic, suggesting that they influence visual mate choice behavior by affecting expression of one or more nearby genes. We searched for such differentially expressed (DE) genes using bulk RNA-seq data from 293 retina, optic lobe (including lamina and medulla), and central brain samples from male and female *H. c. alithea* and *H. c. galanthus* at seven developmental stages (S3 Table). Pure yellow- and white-winged *H. c. alithea* were collected by setting up crosses between virgin individuals from a butterfly breeder that were homozygous for the yellow or white color alleles. We then identified genes that were DE between yellow and white males in each tissue at each developmental stage using DESeq2 or across development using maSigPro (i.e., genes with different temporal expression profiles) (Fig 3) [51–53]. This approach therefore uses wing color as a proxy for preference, which could reduce power to detect differential expression associated with differential mate choice behavior. Altogether, we found that 1,591 genes were DE between yellow and white *H. c. alithea* males in at least one tissue, with the majority (78%) DE in one tissue at one stage (Fig 3; S4–S7 Tables).

Importantly, seven genes were DE and near to the top choice GWA variants. Three *K* locus genes were DE: the basal transcription factor gene *tFIIB*; *herzog*, a cell membrane-associated phosphatase expressed in glia and required for eye development in *Drosophila* [54]; and *alsin-2*, a gene required for proper synapse development and maintenance in *Drosophila* and vertebrates (Figs 2 and 3C) [55,56]. *herzog* and *alsin-2* differential expression occurred in mid- to late-pupal optic lobe and central brain, with significantly higher expression in yellow *H. c. alithea* males than in white *H. c. alithea* or *H. c. galanthus* (Fig 3C). In addition to the *K* locus, 4 out of the 10 genes found within 50 kb of the top choice variants on chromosomes 7, 9, and 11 were also DE. The orthology and functions of three of these genes, *Hcay209001o.g78*, *Hcay211001o.g567*, and *Hcay211001o.g572,* were unknown*.* However, the chr9 gene *mind the gap* was DE in late-stage retina and optic lobe and is known to be involved in synapse assembly throughout the *Drosophila* nervous system. In particular, *mtg* is required for targeting inner PR neurons (fly R7 and R8) to the lamina and medulla (Fig 3C) [57,58]. We therefore predict that genetic variation near these genes alters their expression patterns in the developing visual systems of white and yellow *H. c. alithea* males, resulting in divergent mate preferences.

We attempted to gain deeper insight into the developmental pathways and gene networks that were affected by differential expression between *H. c. alithea* males. We expected that networks involved in mate choice behavior variation would be enriched for DE genes while those with conserved functions would be deficient. We reconstructed the *H. c. alithea* gene co-expression network, inferred potential functions of co-expressed gene modules using Gene Ontology enrichment, and identified modules that were significantly enriched or deficient with DE genes (S6 Fig). Six of 40 gene modules were significantly enriched with DE genes (S6 Fig). The most significantly enriched modules were small ( <100 genes), but involved in vesicle-mediated transport and regulation of signal transduction, suggesting that gene expression variation between *H. c. alithea* males primarily affects the strength or patterning of synaptic communication rather than general patterns of neural development (S6 Fig). These enriched modules included *mind the gap*, *herzog*, and 1:1 orthologs of known *Drosophila* neural development genes such as *Rich*, *cinnamon*, and *ninaA* that are essential for synapse development and responses in fly R7 and R8 PRs, PR maintenance, and PR construction, respectively, that will be important to explore in future studies (Fig 3; S4–S7 Tables) [41]. Future work will be aimed at determining the precise cell types that these candidate genes are expressed in and where in the developing visual system they exert their effects. Altogether, the GWA and DE analysis results point to a handful of strong candidate genes for *H. c. alithea* mate choice variation that will serve as a critical jumping-off point for future experimental studies.

### Variability in UV photoreceptor spectral sensitivity

Gene expression analysis uncovered differential expression throughout the eye and brain, suggesting many possible neural loci where variability could be important for modulating courtship preference behavior (Figs 1D and 3). We opted to begin our investigation on the neural correlates of courtship behavior in the eye for several reasons. First, gene modules that were most affected by differential expression contained many genes involved in eye development, including *herzog* and *mtg.* Second, the peripheral nervous system is known to be an evolutionarily labile hotspot for modification [8–11]. Finally, the *Heliconius* eye displays extreme diversity across the genus [28,29], but there is limited knowledge about the specific organization of the eye across *H. cydno* butterflies (but see [59]). Thus, understanding the potentially variable sensory information available to the butterflies was an important first step towards describing the circuits associated with this vision-based courtship behavior.

To characterize the encoding of sensory information in the retina, we measured the spectral tuning and response properties of single PRs using sharp intracellular electrodes while presenting brief flashes of monochromatic light. In addition to recording from 5 groups of *H. cydno* butterflies separated by species and wing color, we also included *H. melpomene* as a closely related outgroup. Based on previous results [59] and analysis of the data presented here, we observed a strong sexual dimorphism where male eyes varied with species and wing color while female eyes did not. We therefore grouped all females together, regardless of species or wing color, and treated them as a single group separate from males.

**Fig 4:**
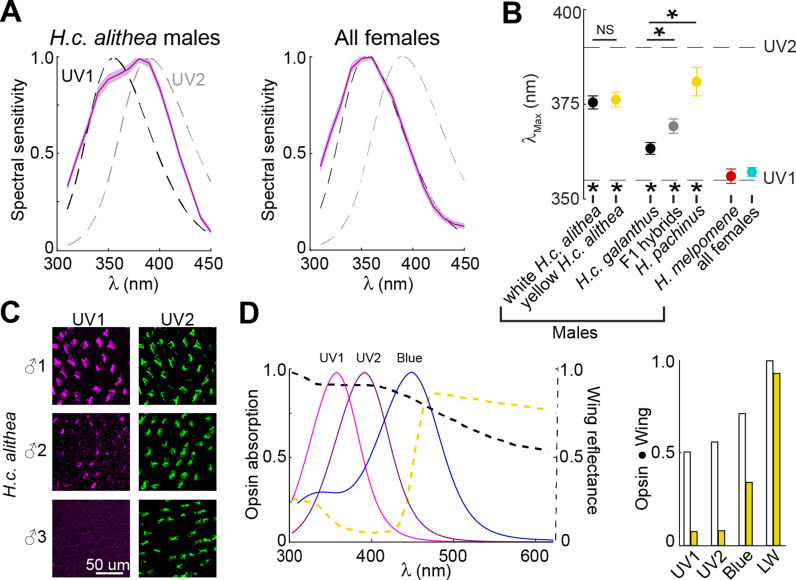
Variable co-expression of UV1 and UV2 within single photoreceptors cannot explain mate choice variability. (**A**) Spectral sensitivity (mean ±  SEM) of UV photoreceptors measured for *H. c. alithea* males (left) and all females (right). Dotted lines indicate the expected sensitivity for UV photoreceptors expressing either the UV1 or UV2 opsin. (**B**) *λ*_Max_ was estimated for each cell with a rhodopsin tuning template and separated into groups based on species, sex, and wing color (*n* =  43 cells/12 individuals, 40/14, 18/9, 19/9, 8/4, 30/16, 22/10). Asterisks above indicate significant differences between groups assessed using pairwise GLME models (*t*-statistic, *p* <  0.05) with negligible random effects due to grouping by individual animal, and asterisks below indicate a significant difference from the expected tuning of both UV1 and UV2 (*t* test, *p* <  0.05 with Bonferroni correction). (**C**) Representative anti-UV1 and anti-UV2 antibody stains in three representative white *H. c. alithea* males showing consistent expression of UV2 and variable expression of UV1 across individuals. Additional stains can be found in reference [59], which comprehensively characterizes this variation. (**D**) Overlay of predicted opsin absorption from template tuning curve and wing reflectance (left) and normalized convolution between the two (right) shows that differences in spectral tuning cannot explain differences in courtship preference. The data underlying this figure can be found in Dryad repository dryad.z8w9ghxjz.

Across all butterflies, we recorded from a total of 503 PRs with spectral sensitivities that segregated into five classes broadly consistent with cell types previously described in other *Heliconius* species [29,39]. These included UV sensitive, blue sensitive, and three types of LW sensitive PR (Figs 4 and S7). The three types of LW sensitive comprised a green sensitive cell type that fit the expected tuning of the LW opsin, a red-shifted variant that is likely derived from a combination of the LW opsin and a red screening pigment, and a broadband sensitive variant that is likely derived from co-expression of the blue and LW opsin (S7 Fig) [39]. For each PR, we estimated the wavelength of peak sensitivity (*λ*_Max_) by fitting the measured spectral sensitivity with a rhodopsin tuning template [60]. The spectral tuning of blue and LW sensitive PRs did not vary across groups (S7 Fig).

In contrast, the spectral sensitivity of UV sensitive PRs varied significantly across groups, with *λ*_Max_ ranging continuously between 345 and 404 nm across all cells (Figs 4A, 4B, and S8). For *H. melpomene* males and all females, *λ*_Max_ was not significantly different from the expected tuning of UV1. For all *H. cydno* males; however, UV spectral tuning was significantly different from the expected tuning of both UV1 and UV2 opsins (*p* <  0.05). Within each group, the distribution of *λ*_Max_ was unimodal (S8 Fig), so this variability cannot be explained as differences in the proportion of cells expressing UV1 or UV2. Instead, we previously showed that single PRs can co-express both UV1 and UV2 [59], and the relative expression of each opsin within a cell may function to tune the specific *λ*_Max_. Consistent with this interpretation, we observed substantially more within-group variability in UV cell *λ*_Max_ values than blue cell *λ*_Max_ values (S8 Fig). This effect was most prominent in *H. c. alithea*, where antibody staining revealed that UV2 staining was strong in every male, while UV1 staining qualitatively varied from strong to weak to absent (Fig 4C).

Across all groups, we observed a weak correlation between *λ*_Max_ and male courtship preferences (Fig 4B). Groups with increasing preference for yellow females tended to have *λ*_Max_ at longer wavelengths, indicative of stronger UV2 expression. To compare UV spectral tuning across groups, we used a generalized linear mixed-effects (GLME) model to account for recording from multiple PRs in some individuals. This model showed that predicted male courtship preference (white, yellow, or none) was a significant predictor of *λ*_Max_ (*p* <  0.001). However, this apparent relationship is unlikely to play a causal role in preference for white or yellow females for two main reasons. First, a GLME specifically comparing *λ*_Max_ in white and yellow *H. c. alithea* revealed no significant differences (*p* =  0.98), in contrast to the observed behavioral differences (Figs 1B and 4B). Second, the primary difference between white and yellow wings is the presence or absence, respectively, of reflectance below ~ 420 nm (Fig 4D). Convolving wing reflectance measurements with opsin absorption showed that white wings strongly excite while yellow wings weakly excite UV PRs regardless of the specific *λ*_Max_ (Fig 4D). This means that wing reflectance evokes nearly identical primary sensory responses in the eye of every butterfly, despite differences in UV spectral tuning. Together, these results show that courtship behavior cannot be explained by small changes in sensory reception, instead likely requiring modifications to downstream neural circuits that can modulate the cognitive perception of the two distinct wing colors.

### Inter-photoreceptor UV inhibition correlates with male courtship preferences

**Fig 5:**
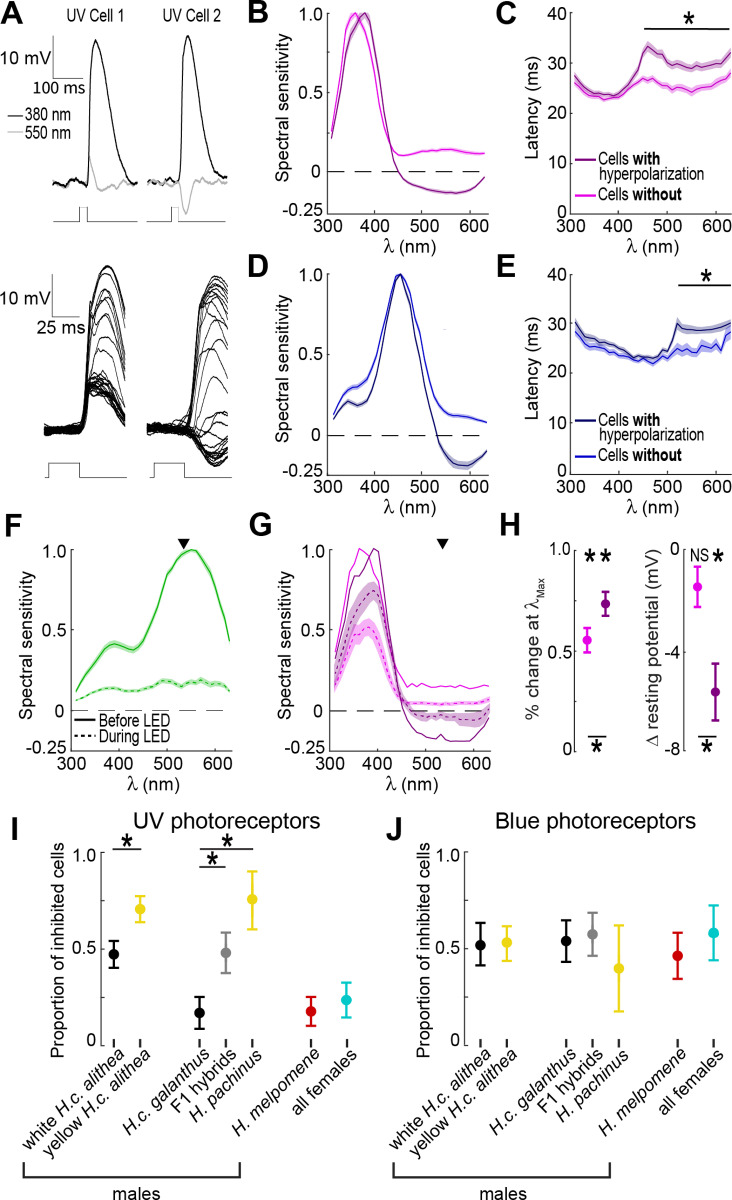
Inter-photoreceptor inhibition onto UV photoreceptors correlates with male courtship preference. (**A**) Example trials for two different UV photoreceptors responding to two different wavelengths (top). Note the difference in polarity for the response to 550 nm (bottom). Zoomed in view of the response to every tested wavelength for the same two cells. (**B**) Tuning curves (mean ±  SEM) for UV photoreceptors separated into groups with (*n* = 76) and without (*n* = 104) hyperpolarizing responses to long wavelength stimuli. (**C**) Response latency for the UV photoreceptors shown in panel B, with the asterisk indicating significant differences between the two cell types at each wavelength (*p* <  0.05). (**D**) Same as B, but for blue sensitive photoreceptors (*n* = 67, 59). (**E**) Same as C for blue sensitive photoreceptors. (**F**) Average tuning curve (mean ±  SEM) for green sensitive photoreceptors (*n* =  40) before and after turning on an adapting, background LED (535 nm, indicated by arrow). (**G**) Same as D, but for UV photoreceptors separated into cells with (*n* =  19) and without (*n* =  18) hyperpolarizing responses. Error bars are omitted from the before tuning curves for clarity. (**H**) Quantification of the change in response magnitude (left) and baseline resting potential (right) after turning on the LED. Asterisks above indicate a significant change from before the LED and asterisks below indicate significant differences between groups (*t*-tests, *p* <  0.01). (**I**) Proportion of UV photoreceptors with inhibition ( ±SEM) across groups (number of cells =  43, 40, 18, 19, 8, 30, 22, number of individuals =  12, 14, 9, 9, 4, 16, 10). Asterisks indicate significant differences between groups (pairwise generalized linear mixed-effects models, *t*-sta*t*istic, *p* <  0.01). Random effects based on individual identity were negligible in all comparisons. (**J**) Same as I for blue photoreceptors (number of cells =  21, 30, 22, 21, 5, 15, 12, number of individuals =  10, 13, 10, 9, 3, 12, 8). The data underlying this figure can be found in Dryad repository dryad.z8w9ghxjz.

Our PR recordings also showed variability in an established physiological signature of inter-PR inhibition that could potentially provide a more significant change in the cognitive perception of wing color (Fig 5). Insect PRs typically depolarize in response to light, but for a subset of UV and blue sensitive PRs, long wavelength stimuli instead evoked a hyperpolarizing response (Fig 5A–5E). These hyperpolarizing responses have now been detected in several butterflies and likely correspond to color-opponent inhibitory input from other PRs with different spectral sensitivities [23–25,41–43]. To assess whether the hyperpolarization we observed might also be indicative of inhibition from LW sensitive PRs, we first measured response latencies (Fig 5C, 5E). Compared to the response latency at *λ*_Max_, these hyperpolarizing responses were delayed by an average of 5.4 ±  0.59 ms, consistent with time delays associated with monosynaptic inhibition. These latency differences were consistent over two log units of stimulus intensity (S9 Fig). For PRs without hyperpolarizing responses, the latency of the small depolarizing responses to long wavelength stimuli were not significantly different from *λ*_Max_.

Complementary to latency measurements, we also recorded from UV PRs in the presence of an adapting, background 535 nm LED. This LED, two times more intense than the monochromatic stimuli, persistently activated LW sensitive PRs, reducing their responses to monochromatic stimuli to 20.9 ± .02% of the baseline response and raising their baseline potential by 5.6 ±  1.2 mV (Fig 5F). The dark-adapted resting potential did not differ between the UV cells with and without hyperpolarization (−52.7 ±  1.6 mV, *p* =  0.84), but the LED differentially affected them. Consistent with inter-PR inhibition, the LED significantly decreased the resting potential of UV PRs with hyperpolarizing responses (−5.6 ±  1.1 mV, *p* <  0.001), while the resting potential of UV PRs without hyperpolarizing responses did not change significantly (−1.4 ±  0.8 mV, *p* =  0.10) (Fig 5H). The LED also decreased response magnitude at *λ*_Max_ for all UV cells, but this decrease was significantly larger for UV PRs without inhibition (Fig 5G, 5H). Finally, we observed differences across PRs in the temporal duration of a response to the short monochromatic flashes (S10 Fig). These durations were significantly shortened by the presence of the LED. Together, the latency measurements and LED experiments show that a subset of UV and blue PRs likely receive monosynaptic inhibitory input from LW sensitive PRs.

Comparing the proportion of inhibited cells across groups revealed that variability in UV PR inhibition, but not blue, was strongly correlated with male courtship preferences (Fig 5I, 5J). For blue cells, we observed no differences across groups, with 53.2 ±  4.5% showing evidence of inhibitory input (Fig 5J). For UV cells, however, courtship preference was a significant predictor of long wavelength inhibition in a GLME (*p* <  0.001), as we observed increasing proportions of inhibited UV PRs in groups that increasingly prefer yellow females (Fig 5I). Few UV cells showed evidence of inhibition across *H. melpomene* males and all females (19.2 ±  5.5%, 10 of 52 cells). For *H. cydno* males, only a limited number of cells showed evidence of inhibition for white preferring *H. c. galanthus* (16.7 ±  8.8%, 3 of 18 cells), while 70.8 ±  6.6% (34 of 48 cells) across *H. pachinus* and yellow *H. c. alithea* were inhibited. F1 hybrids and white *H. c. alithea*, both of which show no behavioral preference, had intermediate rates of inhibition (46.8 ±  6.3%, 29 of 62 cells). Importantly, in contrast to our findings on UV PR spectral sensitivity (Fig 4B), a GLME model specifically comparing white and yellow *H. c. alithea* also revealed a significant fixed effect based on wing color (*p* =  0.008, Fig 5I). This difference was especially meaningful because these butterflies are derived from a single interbreeding population and are genetically indistinguishable outside of the *K* locus (S11 Fig). This result strongly suggests that the gene(s) controlling UV inhibition variation in this subspecies are tightly linked to the wing color gene *al-1*, i.e., within the *K* locus. UV inhibition in *H. c. galanthus* was also significantly different from both its sister *H. pachinus* (*p* =  0.001) and their F1 hybrid offspring (*p* =  0.004). Together, these results suggest that modulation of UV PR inhibition could potentially provide the significant computational change necessary for shifting male preference for white versus yellow females.

## Discussion

The survival and reproduction of an animal depends on its ability to adapt its behavior to its environment, either through learning or through evolution across generations. The evolution of novel, adaptive behaviors requires that they must be heritable (i.e., have a genetic basis) and that the benefit of the underlying genetic and neural changes outweighs any potentially detrimental, pleiotropic effects. While the evolvability of the periphery typically refers to changes in receptor sensitivity like we observed in the spectral tuning of UV PRs, larger computational changes mediating more complex behaviors (like color preference in courtship) are thought to require changes to central circuits. Our work unites these ideas, and adds to a growing number of studies identifying changes in the periphery associated with changing behavior, from opsin expression variation in cichlid fishes to peripheral motor neurons underlying fly song evolution [13,61–64]. However, the gaps between heritable genetic variation, development, and peripheral sensory systems remain difficult to fill.

### The genetic architecture of mate choice variation in *H. cydno*

Our integrated bottom-up genetic and top-down neurobiological approaches helped us begin to narrow these gaps in the evolution of *Heliconius* mate choice behavior. We identified three strong correlates with *H. cydno* mate choice variation: genetic variation in the *K* locus and a handful of other loci across the genome; expression variation of genes at these loci; and variation in inter-PR inhibition onto UV-sensitive PRs. We hypothesize that the proximate cause of *H. cydno* mate choice variation is genetic variation in the *K* locus that directly affects expression of a small number of genes, either by directly altering *cis*-regulatory element sequences or their interactions with nearby gene promoters. GWA, RNA-seq, and ortholog functions each suggest that *alsin-2* and *herzog* may be those genes. *Alsin-2*, in particular, is a Rho GEF known to be required for synapse development and function in both vertebrates and flies [56], and we observed differential expression of *alsin-2* in mid-pupal optic lobes and central brains between yellow and white males. It is conceivable that allelic variants of *alsin*-2 and/or *herzog* directly regulate the differential development or distribution of inhibitory synapses onto UV PRs in yellow- and white-preferring males. Alternatively, differential expression may alter cell type composition between yellow- and white-preferring males that could differentially wire their peripheral visual systems. Similar arguments could be made for genes near the GWA peaks on chromosomes 7, 9, and 11 including *mtg*. However, future work that directly visualizes candidate gene expression in the developing visual system, the locations and density of inhibitory synapses onto UV PRs, and experimentally tests the effects of candidate locus genes on mate choice behavior will be critical for filling the remaining gaps between these three layers of variation.

Alternatively, or in addition to their direct effects, differential expression of these *K* locus genes could have cascading effects on gene expression that ultimately affect the development and/or maintenance of visual circuits underlying mate choice behavior. Hundreds of genes were DE between white and yellow *H. c. alithea* male central brains, optic lobes, and retinas despite samples being harvested from a single population. However, DE genes were concentrated in networks involved in synaptic communication and signal transduction, consistent with the variation in UV PR inhibition we observed in the eye (Figs 3–5 and S6). In particular, multiple genes involved in axon guidance and synapse formation in the retina were DE in the *H. c. alithea* retina and optic lobe. Future experiments integrating single-cell or spatial transcriptomics approaches will be needed to fully characterize how these networks operate, but these results provide a necessary framework to build upon. For example, one hypothesis is that differential expression in retina of *K* locus genes like *tFIIB* may drive differential expression of *Rich, cinnamon,* and *ninaA* and other genes that directly impact the development of inhibitory synapses onto UV PRs that we observed in *H. c. alithea* (Fig 3). Thus, while the precise links between differential expression and differential development of these synapses remains unknown, a few strong candidate genes and networks are ripe for experimental investigation.

### Observed variability in UV opsin tuning cannot explain mate choice

For our physiology recordings, we first observed significant variation in the spectral tuning of UV PRs that weakly correlated with courtship preferences (Fig 4). However, the lack of a difference between white and yellow *H. c. alithea* indicates that UV opsin expression is likely not playing a causal role in courtship preferences. Instead, our data are consistent with an existing hypothesis about a different evolutionary role of UV2 in *Heliconius* behavior [40]. *Heliconius* uses a genus specific yellow pigment (3-OHK) for wing color that is distinct from the yellow pigment used in sympatric species that often mimic the wing patterns of unpalatable *Heliconius* butterflies. Previous research has demonstrated that, compared to UV1, the spectral tuning of UV2 significantly enhances the discriminability of these two pigments with subtly different reflectance spectra [65]. Thus, for butterflies that will court yellow females—including those with a yellow preference and those with no preference—expression of UV2 may confer an ecological advantage as they will be better able to discriminate between a yellow *Heliconius* female and a non-*Heliconius* mimic. Butterflies that do not actively approach and court yellow females would not face this ecological pressure to discriminate between the yellow pigments, and in these groups we predominantly detected UV1 expression.

The convolution between wing reflectance and opsin absorption (Fig 4D) further highlights that no relatively simple shift in opsin tuning and sensory reception can explain courtship preferences. Whereas the tuning shift from UV1 to UV2 can improve discriminability of two subtly different yellow pigments, white and yellow wing colors are highly discriminable, even to humans who lack a UV PR entirely. Preference necessitates discrimination, and the strong behavioral preference of *H. c. galanthus* and *H. pachinus* indicates that both can readily discriminate the two wing colors despite divergent UV spectral tuning that approaches the tuning of UV1 and UV2, respectively. The convolution analysis confirms this intuition (Fig 4D), as it shows that white wings should evoke strong and similar responses in UV PRs regardless of the specific *λ*_Max_, while yellow wings should evoke almost no response. Thus, the sensory information transmitted to the optic lobes following transduction of a white or yellow wing pattern stimulus into a PR response should be nearly identical for every butterfly we examined. Together, these results suggest that the underlying neural mechanisms that facilitate differences in male courtship behavior act on downstream cognitive perception of color rather than sensory reception and stimulus discrimination.

### Inter-photoreceptor inhibition as a flexible motif in peripheral vision

Our physiology recordings additionally revealed evidence of inhibitory inter-PR synapses, a finding that has now been observed in *Drosophila* and more than 10 butterflies [23–25,41–43], suggesting a common circuit motif across insect visual systems. The inhibition we observed closely resembles PRs termed U+G−, B+G−, and G+R− in other Nymphalid species [24,25]. Many of these previously reported G+R− PRs showed evidence of co-expression of the blue opsin, in agreement with the broadband sensitivity of the G+R− PRs we recorded from here (S7 Fig). For blue PRs, we detected evidence of inhibition in approximately 50% of the cells, and this proportion did not vary across the seven groups we analyzed. For UV PRs, in contrast, we found that the proportion of inhibited cells correlated well with male courtship preferences, including differences between white and yellow *H. c. alithea*. Additionally, while U+G− PRs have been recorded in other Nymphalid species [25], the relative lack of UV inhibition we observed in *H. melpomene*, all females, and other *Heliconius* species [28,39] further points towards UV inhibition being unique to *H. cydno* males that court yellow females.

The potential for variability in inter-PR inhibition of UV cells to influence mate choice behavior suggests that more features of the evolutionarily labile peripheral nervous system than just receptor sensitivities may be subject to rapid evolutionary change. However, with limited data on how color and courtship computations are implemented in the optic lobes and central brain of *Heliconius* or any other butterfly, it is difficult to model and infer how differences in inter-PR inhibition could causally affect behavior in a satisfactorily constrained manner. Existing color-space models focus on stimulus discrimination based solely on receptor sensitivities (e.g., [66]), but cannot model cognitive preferences or incorporate specific changes in circuit connectivity. Anatomy in *Papilio xuthus* shows that these inter-PR synapses occur in the axons in the lamina, which could allow for potentially large and nonlinear effects on how graded synaptic transmission in these UV PRs functions [23,43].

With these caveats, our working hypothesis for how UV PR inhibition could causally contribute to mate choice behavior is based on two observations. First, mate choice is based on a cognitive choice rather than sensory reception and discrimination (Fig 4D, see above). Second, yellow is the ancestral wing color in *H. cydno* butterflies [26]. Thus, rather than males making a de novo choice between white and yellow females, one might instead view yellow as the ‘default’ color preference. With yellow attraction as the ancestral state, the brain would be broadly organized to promote courtship towards yellow females rather than other colors such as the red wings of *H. melpomene*. Making white wings attractive would then mean overriding an innate attraction towards yellow.

Thus, based on these two ideas, we speculate that input from UV PRs into courtship circuits has a generally positive courtship valence in *Heliconius*. That is, when UV PRs respond to a wing color pattern, it might increase the perceived attractiveness of the detected female. Since yellow wings largely lack UV reflectance, suppressing UV signaling via inter-PR inhibition could function as one of potentially many neural loci to make yellow females attractive to the ancestral *H. cydno* butterfly. When white wings with strong UV reflectance evolved, releasing this inhibition and allowing stronger UV signals to propagate into the brain could then serve to make the white wings more attractive. Existing color opponent computations, homeostatic mechanisms, or circuit modifications from DE genes throughout the brain could plausibly function to suppress the attractiveness of yellow females to white males. Testing this hypothesis and understanding the mechanisms controlling courtship in these butterflies will entail substantial future work elucidating the associated central brain circuits.

### Cue and preference co-evolution via physically separate loci

Finally, our results help clarify the mechanisms that underlie co-evolution of wing color and preference. Co-evolution between color and preference in *H. cydno* is mediated by genetic coupling between two physically separate, but linked loci rather than a single pleiotropic gene or genome structural variation. Theory predicts that speciation should be rare when preference and cue are controlled by separate loci because recombination should quickly break down association between the two traits [50,67,68]. However, the mechanism of coupling we observed in *H. cydno* may be common, especially in *Heliconius* [21]. Coupling may be partly caused by assortative mating itself [69,70]—that is, assortative mating will automatically cause an increase in frequency of the coupled alleles—but is likely enhanced in *Heliconius* by natural selection against locally rare aposematic wing colors, which eliminates individuals with mismatched color and preference alleles [71]. *Heliconius* have rapidly evolved myriad wing color patterns, and genetic coupling should entail similarly rapid adaptations of the nervous system. Thus, genes important to the functional organization of an evolutionarily labile periphery may play an important role in facilitating the initial stages of the speciation process.

## Materials and methods

### Animals

We used butterflies from four different taxa. The *H. c. alithea* used in the preference and color GWA analyses were previously tested for courtship behavior in Ecuador in 2008 by Chamberlain and colleagues [16]. These butterflies were tested for their preference, then the bodies stored in 100% ethanol at −80°C until genomic DNA extractions (see below) [16]. For all other experiments, butterflies were housed in greenhouse breeding colonies at the University of Chicago that were regularly supplemented with new individuals. Adults were fed Bird’s Choice artificial nectar *ad libitum* and supplied with blooming *Lantana* as an additional source of nectar and pollen. *H. c. galanthus* and *H. melpomene* pupae were obtained from El Bosque Nuevo in Costa Rica, and *H. c. alithea* from *Heliconius* Butterfly Works in Ecuador. *H. pachinus* and F1 *H. c. galanthus* × *H. pachinus* hybrids were bred in Panama and adults were transported to Chicago for experiments. Collection, rearing, import and export were done under permits from Ecuador, Panama, Costa Rica, and the United States of America (USA).

### *Heliconius cydno alithea* (yellow) genome assembly and annotation

We isolated DNA from thorax of a single adult yellow *H. c. alithea* female using the QIAGEN Genomic-tip 20/G following the manufacturer’s instructions with the following modifications: tissue was incubated at 50°C shaking at 800 rpm overnight in lysis buffer. We used 4 μg of this high molecular weight DNA as input to Oxford Nanopore Technologies (ONT) ligation library preparation kit SQK-LSK 110. We prepared libraries following the manufacturer’s instructions with modifications based on the protocol found here: https://www.protocols.io/view/dna-extraction-and-nanopore-library-prep-from-15-3-bp2l6n3kzgqe/v1. End-repair was performed at 20°C for 1 h, dA-tailing was performed for 30 min, ligation was performed for 1 h at room temperature, and all bead elution steps were allowed to proceed for 1 h at room temperature. Finally, we used the PacBio SRE XS kit to remove <10 kb fragments from the final libraries.

Final libraries were sequenced on an ONT MinION with version 9.4.1 flow cell. We performed basecalling using Guppy and the super accurate basecalling model in dna_r9.4.1_450bps_sup.cfg supplied with the basecaller. We adopted a strategy similar to Steward and colleagues (2021) to perform genome assembly [72]. We generated the initial draft assembly using Flye 2.9 [73] with estimated genome size of 282 Mb (based on GenomeScope estimate https://github.com/schatzlab/genomescope) and Shasta 0.10.0 (https://github.com/chanzuckerberg/shasta) with default parameters. The Flye assembly and Shasta assembly were polished with two rounds of racon 1.5.0 (https://github.com/isovic/racon) and one round of medaka 1.8.1 with ONT reads, and then purged to remove duplicate scaffolds (typically uncollapsed allelic variation) using purge_dups (https://github.com/dfguan/purge_dups). Finally, the duplicate scaffolds were merged together with quickmerge (https://github.com/mahulchak/quickmerge) and purged using purge_dups.

To simplify comparisons across species, we scaffolded *H. c. alithea* contigs to the *Heliconius melpomene* v2.5 chromosome-level assembly using RagTag [74] and renamed *H. c. alithea* scaffolds to match. Finally, we identified and soft-masked repeat sequences using RepeatModeler and RepeatMasker [75,76]. The genome sequence and annotation used in this study can be found in Dryad repository dryad.z8w9ghxjz. The final genome assembly comprised 310 scaffolds spanning 294 Mb, with 287 Mb assigned to *H. melpomene* chromosomes. BUSCO v5 analysis showed the *H. c. alithea* genome contained 97.7% complete (97.3% single-copy, 0.4% duplicated), 0.4% fragmented, and 1.9% missing OrthoDB v10 Endopteryogota (2,124 single-copy orthologs) SCOs.

We annotated *H. c. alithea* scaffolds using EvidenceModeler 1.1.1 [77]. We first assembled the *H. cydno* transcriptome de novo using RNA-seq data generated by Walters and colleagues [78], Nallu and colleagues [79], and Rossi and colleagues [80] using Trinity v2.10.0 [81]. RNA-seq data was also mapped to using STAR 2.6.1d [82], and the resulting alignments used to generate genome-guided assemblies using Trinity and StringTie 1.3.1 [83]. We combined de novo and genome-guided assemblies using PASA [84]. Evidence for protein-coding regions came from mapping the UniProt/Swiss-Prot (2020_06) database and all Papilionoidea proteins available in NCBI’s GenBank nr protein database (downloaded 6/2020) using exonerate [85]. We identified high-quality multi-exon protein-coding PASA transcripts using TransDecoder (transdecoder.github.io), then used these models to train and run Genemark-ET 4 [86] and GlimmerHMM 3.0.4 [87]. We also predicted gene models using Augustus 3.3.2 [88], the supplied *heliconius_melpomene1* parameter set, and hints derived from RNA-seq and protein mapping above. Augustus predictions with > 90% of their length covered by hints were considered high-quality ab initio models. Transcript, protein, and ab initio data were integrated using EVM with the weights in S8 Table.

Raw EVM models were then updated twice using PASA to add UTRs and identify alternative transcripts. BUSCO v5 analysis of the final annotated protein set showed it contained 94.8% complete, 1.8% fragmented, and 3.4% missing OrthoDB v10 endopteryogta SCOs (*n* =  2124). Gene models derived from transposable element proteins were identified using BLASTp and removed from the annotation set. Functional annotations were applied to the final annotation set using eggNOG mapper v5 [89]. The final annotation comprises 18,763 protein-coding genes and 30,325 transcripts. We identified 1:1 orthologs to *Drosophila melanogaster* proteins using reciprocal BLASTp, assigning orthologs only to those genes where the top hit was identical between the two directions (i.e., Hca →  Dmel AND Dmel →  Hca). Gene annotations, eggNOG results, and Drosophila orthologs are supplied in the Dryad repository dryad.z8w9ghxjz.

### *Heliconius cydno alithea* genome re-sequencing and variant calling

Genomic DNA was isolated from thorax of 113 *H. c. alithea* males studied by Chamberlain and colleagues [16] using chloroform extractions (S1 Table; BioProject PRJNA802836). We re-sequenced all individuals with multiple courts, plus a number of males with just a single court, that produced high-quality genomic DNA. This yielded a subset of 113 males from the 175 included in the original study. Illumina paired-end libraries were constructed using the KAPA Hyper Prep Kit (KAPA Biosystems) or Nextera Library Prep Kit (Illumina) and sequenced to ~ 15× using 2 × 100 bp on an Illumina HiSeq2500 or 4000 at the University of Chicago Functional Genomics Facility.

Low-quality regions and adapters were trimmed from raw reads using Trimmomatic before mapping to the *H. c. alithea* reference using bowtie2 v2.3.2 with default settings except *--very-sensitive-local* [90]. We then marked PCR duplicate reads with Picard and realigned around putative indels using the Genome Analysis Toolkit (GATK) 3.8 [91,92]. SNP and indel calling was performed using the HaplotypeCaller and GenotypeGVCFs module in GATK 4.3.0 with the heterozygosity priors set to 0.01 for both SNPs and indels. Scripts and variant calls in PLINK bed/bim/fam format can be found in the Dryad repository dryad.z8w9ghxjz.

### Genomics analyses

We first pruned our final variant set using PLINK’s (1.90) LD-based pruning and basic allele frequency cutoffs (i.e., *--indep-pairwise 1000 100 0.80 -miss 0.05 -maf 0.05 -hwe 1e-50*) [93]. This step reduces the number of tests required and reduces false positives due to extreme violations of Hardy–Weinberg equilibrium assumed in most statistical computations. This filtered dataset consisted of 8.7 M variants and was used for all subsequent genome-wide analyses, including *F*_*ST*_, LD, and GWA.

To estimate allele frequency divergence between yellow and white *H. c. alithea,* we calculated genome-wide *F*_*ST*_ in 10 kb sliding windows (2 kb step) using VCFtools 0.19 [94] and Weir and Cockerham’s method [95] (S11 Fig).

### LD and searches for recombination suppression mechanisms

We calculated empirical LD decay for Fig 2D using a random sample of 600 million pairs of variants from the *K* locus and PLINK 1.90. We then summarized values into 500 bp bins and plotted mean and 95%CI values for each bin in R. Pairwise LD between the top color and preference variants in the *K* locus (Fig 2) was also calculated using PLINK 1.90 using the *--ld <fwc_snp> <choice_snp>* option and parsing the results.

Recombination rates are negatively correlated with the density of repeat elements in a variety of organisms [96]. We tested if increased pairwise LD between the color and preference GWA peaks may be influenced by repeat density in the *K* locus by calculating the density of repeat elements across the locus using RepeatMasker predictions. We found no evidence for increased repeat density between the color and preference peaks, and somewhat decreased density around *K* relative to genome-wide levels (S4 Fig). This result did not change when we limit to just putative transposable elements.

### Genome-wide association analysis for male mate choice

Details on the collection and initial analyses of the male courtship data are found in the original Chamberlain and colleagues (2009) publication [16]. Raw courtship data, scripts to recapitulate the published analysis, and an R notebook detailing QC and exploratory analyses are also provided in Dryad repository dryad.z8w9ghxjz. Following [16], we performed GWA for male mate choice using genotypes and courtship data from the 113 re-sequenced males described above. This dataset included 1,529 choices performed by 57 yellow and 56 white males. We used GMMAT 1.4.2 [97] to perform the GWA, modeling variant effects with a generalized linear mixed model including “contrast.yt” and variant genotype as fixed effects and genetic relatedness (GRM), trial, and male identity as random effects. That is,


$$logit\left(choice\right)\sim intercept+contrast.yt+genotype+\left(1|male\right)+\left(1|trial\right)+\left(1|GRM\right)+error$$

Where errors were modeled using the binomial distribution. We used Wald statistics to test for association. The “contrast.yt” term, the presence of a yellow female with a black triangle (*Ac* patch) on her wings, was found to be a significant predictor of male choice by Chamberlain and colleagues (2009) and here (Dryad dryad.z8w9ghxjz/gwas Section 2). The “trial” term captures the unique set of males and female models present in a cage within a certain trial period [16]. The GRM was calculated in GEMMA 0.98.5 [98]. We identified FDR cutoffs using the Benjamini–Hochberg method [46] and raw Wald test *p*-values from all 8.7 million tests.

GWA for male forewing color was performed using GEMMA 0.98.5, including just the GRM as a random effect. We identified FDR cutoffs using the Benjamini–Hochberg method and raw Wald test *p*-values from all 8.7 million tests.

The appropriateness of each approach was assessed using the genomic inflation factor (λ) and quantile–quantile (Q–Q) plots (S1 Fig; Dryad dryad.z8w9ghxjz). is the ratio of the median ^2^ test statistic in the GWA analysis to the expected median χ^2^ test statistic. Under the assumption that few variant sites contribute to the phenotype of interest, λ should be close to 1. λ values greater than 1.05 or 1.10 indicate *p*-value inflation caused by, e.g., residual population structure. λ values are shown in Fig 2. Q–Q plots for each analysis are shown in S1 Fig.

Marginal effects and confidence intervals of the top *K* locus, chr7, chr9, and chr11 variants were calculated using *lme4::glmer* and the *margins* package [99].

### SuSiE-RSS analysis

We performed statistical fine-mapping using SuSiE-RSS 0.12.27 [44,100]. We performed the analysis using all variants from the *K* locus (chr1:14,500,000-16,500,000), plus the top variants ± 500 variants on chromosomes 7, 9, and 11. In total, this included 61,824 variants. We used as input to *susie_rss* the coefficient estimates (*bhat*) and standard errors (*shat*) contained within the GMMAT output. The LD matrix was calculated by converting variant genotypes to numeric using PLINK (*--recode A*), loading the resulting matrix into R, replacing missing genotypes with the mean genotype for that site, and calculating correlation coefficients using the *cor()* function. Sample size was set to 1,529 (the number of courtship events).

### *Heliconius* RNA-sequencing

We aimed to collect RNA-sequencing data from retina, optic lobe, and brain tissue at seven developmental stages in *H. c. galanthus*, white *H. c. alithea*, and yellow *H. c. alithea* males and females in triplicate (see Fig 3). We used controlled crosses between *H. c. alithea* males and females that were homozygous for the top wing color variant, thus ensuring that larvae and pupae from each cross would (if they were allowed to emerge) develop a single wing color. We identified appropriate adults for crosses by clipping a single leg from each individual that emerged from each shipment, extracting DNA from that leg using DNA ExtractALL reagents (Thermo), then performing a custom TaqMan genotyping assay for the top wing color GWA variant using the leg DNA. Only males and females that were homozygous for the yellow or white allele were used to set up “yellow” or “white” crosses. All *H. c. galanthus* individuals were used in *H. c. galanthus* crosses. We set up crosses between multiple males and females in the UChicago greenhouse and provided ample host plants for egg lay. Caterpillars and pupae were maintained in separate small cages for each cross, and individuals were labeled upon pupation to track developmental timing. We collected tissues from one larval stage (final instar purple crawler, ~ 36 h before pupation), five pupal stages (p0: 12–24 h after pupation, p2: 48–60 hap, p4: 96–108 hap, p6: 144–156 hap, and p7: 168–180 hap), and one adult stage (ad: 24–48 h after emergence). Pupal sex was determined using external pupal characteristics (https://www.ucl.ac.uk/taxome/jim/Mim2/heliconius_pupa_sex_difference.html) as well as the presence/absence of testis, which are very prominent in butterflies.

We collected head tissue from purple crawler and p0 pupae because the main neural tissues are small and difficult to separate. We collected retina, optic lobe, and central brain separately for all remaining stages. We dissected individuals in cold PBS and immediately placed dissected tissues into RNAlater (Ambion, USA). Tissues were stored in RNAlater at −80°C until RNA extraction using TRIzol (Ambion, USA). High quality (RIN >  7) RNA samples were treated with Turbo DNAse (Invitrogen, USA), then 1 µg was used as input for poly-A selection and RNA-seq library preparation using the NEBNext Poly(A) mRNA Magnetic Isolation Module and NEBNext UltraII Directional RNA Prep Kit following the manufacturer’s instructions with minor modifications. RNA fragmentation was performed for 10 min at 94 °C. We used the NEBNext Multiplex Oligos for Illumina dual-index adapters to uniquely barcode each sample. Double-sided selection was performed after adapter ligation to enrich for ~ 300 to 500 bp fragments. Final libraries were PCR amplified for 11 cycles. RNA-seq libraries were pooled and sequenced 2 × 100 bp on a NovaSeq 6000 at the University of Chicago Functional Genomics Facility. All sample information can be found in S3 Table and the raw sequencing data downloaded from NCBI BioProject PRJNA1019262.

### RNA-seq analysis

Quantifications, scripts for analysis, and other data objects can be found in Dryad repository dryad.z8w9ghxjz.

#### Quantification and filtering.

We quantified gene expression in each sample using the raw reads, the yellow *H. c. alithea* transcriptome, and salmon v1.9.0 [101]. The whole genome sequence was included as the decoy, and sequence composition, GC, and positional bias corrections were used during quantification. Indexes and quantification used *k*-mer size 31. Gene-level quantifications for all samples were loaded into R 4.2.3 using tximport 1.26.1 [102]. Quantifications were then loaded into a DESeq2 object and library size normalization factors calculated using DESeq2 [52].

Genes with mean expression values less than 50 were considered lowly-expressed and excluded from all analyses (see Dryad dryad.z8w9ghxjz for exploration and further explanation). We used robust PCA on variance stabilized quantifications (*DESeq2::vst*()) to identify outlier samples, analyzing each developmental stage/tissue separately, following recommendations in Chen and colleagues [103]. Specifically, we used the PcaGrid function from the rrcov R package to reduce the data, then excluded samples with score or orthogonal distances greater than 97.5th percentiles of those distances with the sample group being analyzed [104]. These analyses removed 23 outlier samples. After removing these outliers, we visually inspected sample clustering using rPCA and removed 10 additional samples that aberrantly clustered with divergent tissue/stage groups. The final dataset included 260 samples and 10,409 genes. Raw and filtered quantification data can be found in Dryad dryad.z8w9ghxjz.

#### Stage-specific differential expression.

We did not have direct estimates of male preference for individuals used for RNA-seq. We therefore used forewing color as a proxy, as *H. c. alithea* color is highly correlated with mate preference ([16] and here) and we observed a significant physiological difference between white and yellow males in our electrophysiology experiments (Figs 4 and 5). We first identified genes that were DE between white and yellow *H. c. alithea* males at each stage and in each tissue using DESeq2 [52]. We modeled gene expression by group/sex (i.e., yellow females, white females, yellow males, white males), then tested for DEGs between yellow and white male groups. This is equivalent to fitting a more complex model accounting for group, sex, and their interaction. We considered significantly DE genes to be those with FDR <  0.01 after controlling FDR across all 16 stage/tissue comparisons using the Benjamini–Hochberg method [46].

#### maSigPro analysis.

We identified genes with significantly different expression profiles between white and yellow *H. c. alithea* males within each tissue using maSigPro 1.70.0 [51,53]. We used a dispersion parameter (theta) value of 6.0, and modeled gene expression by group and up to a fourth degree polynomial. Genes with significant profiles were identified using the *maSigPro:*:*p.vector* function and an FDR cutoff of 0.05; variable selection was performed using *maSigPro::T.fit* and a *p*-value cutoff of 0.01. To limit false positive rates, only significant genes with fit *r*^2^ values > 0.8 were analyzed further. These cutoffs were chosen to minimize false positive rates, in line with recommendations by Nueda and colleagues [53]. DEGs between yellow and white males were identified as those that have significantly different expression profiles specifically between those two groups.

#### WGCNA and enrichment analyses.

We reconstructed the gene co-expression network using the WGCNA package and the final normalized *H. c. alithea* RNA-seq quantification data [105,106]. Key parameters included the soft power threshold (determined using in-built functions) of 16, use of signed adjacency matrices, and use of the signed Nowick method to create TOMs. Minimum module size was set to 20 genes.

We assessed module gene ontology (GO) enrichment using the eggNOG annotations and the topGO R package [107]. We used Fisher Exact Tests to determine significance of GO enrichment for each module. We further visualized GO enrichment using GO-figure! [108] with the top 50 GO terms associated with each module, plotting only the top 10 term clusters (summarized for three modules in S6 Fig). We assessed module enrichment with DEGs using Fisher Exact Tests and the combined DESeq2 and maSigPro DEG gene sets.

### Analysis of *Drosophila melanogaster* neural gene orthologs

We first identified 1:1 ortholog between *H. c. alithea* and *D. melanogaster* using BLASTp, requiring that genes were reciprocal best BLASTp hits with *E*-values less than 1. We used the protein set from FlyBase release 6.44 for this. This pipeline assigned 5,790 1:1 orthologs. We then used the FlyBase simple search to identify *D. melanogaster* genes associated with the key words “brain”, “optic lobe”, or “retina”. We then cross-referenced this set of 2,596 genes with our set of DEGs during analysis of gene modules (S6 Fig).

### Intracellular electrophysiology

For in vivo recordings, butterflies at least 3 days old were restrained in a custom built collar with heated beeswax. A small hole was cut in the dorsal eye to allow for electrode penetration along the dorsal–ventral axis of the eye and covered with silicone grease to prevent desiccation. A second small hole was cut near the mouthparts and a silver-chloride reference electrode was placed into the anterior portion of the head. The butterfly was then placed on a stage with the eye at the center of a Cardan arm perimeter device to allow for equivalent light stimulation at any spatial location.

PR responses were evoked using monochromatic stimuli ranging from 310 to 630 nm in 10 nm increments (S12 Fig). The light source was a dual Halogen-Deuterium lamp (DH-2000s, Ocean Optics), which was connected to a scanning monochromator (Monoscan-2000, Ocean Optics). Stimulus timing was controlled with an optical shutter (OZ Optics) and focused onto the butterfly eye using a collimator and lens (Edmund Optics). Every component was connected to each other using 1 mm fiber optic cables. Stimulus intensity was calibrated with a photodiode (Newport) and set to 1.5 × 10^15^ photons/cm^2^/s using a variable neutral density filter in a rotational motor (Newport). Recordings were amplified with a 0.1× headstage and high impedance amplifier (AxoClamp 900A, Molecular Devices) and digitized at 10 kHz (DigiData1550, Molecular Devices).

PRs were recorded intracellularly using sharp electrodes made from borosilicate glass on an electrode puller (P-97, Sutter Instruments). Electrodes were pulled to a resistance between 90 and 120 MΩ and filled with 3 M KCl. Recordings were made exclusively from cells in the ventral half of the eye. All PRs responded to white light with depolarizations of at least 30 mV. Stimuli were presented in a random order with 4 repeats per stimulus. Typically, responses were recorded at multiple intensity levels using neutral density filters (Thorlabs). After recording spectral responses, we also presented the wavelength that evoked the maximum response at nine intensity levels that varied over 4 log units of attenuation. These V-Log(I) curves were used to transform the isoquantal spectral responses of the PRs to a spectral sensitivity curve using the Naka–Rushton equation [109]. The wavelength of peak sensitivity was estimated for each cell by fitting its responses with a standard rhodopsin tuning template [60]. To measure response latency, we first measured the mean and standard deviation of the resting potential for 500 ms before the light flash. Onset latency was defined as the time for the response to exceed five times the standard deviation of this baseline.

For experiments with the LED, we used green LEDs with peak tuning at 534 nm and a full width half maximum of 12 nm. Six LEDs were attached to the monochromatic source and had an intensity of 3.2 × 10^15^ photons/cm^2^/s. Spectral responses were recorded from each cell before, during, and after turning on the LEDs. This intensity did not bleach PR responses, as the full response magnitude was typically recovered within seconds of turning off the LED. PRs that did not recover at least 80% of the original response were discarded.

When comparing physiology data across groups of butterflies (Figs 4B, 5I and 5J), we tested for significance using GLME models with a logit link function to account for repeated measures within single butterflies. For each model, butterfly identity was included as a random effect. For each analysis, we first computed significance using courtship preference (white, yellow, or equal) as a fixed effect, effectively grouping together taxa with similar behavior (e.g., F1 hybrids with white *H. c. alithea*). We then conducted a series of models comparing white and yellow *H.c. alithea* and all pairwise comparisons between *H. c. galanthus*, *H. pachinus*, and the F1 hybrid offspring of this pair. For models looking at long wavelength inhibition (Fig 5), we used the normalized response amplitude of a cell at 530 nm for UV cells and 590 nm for blue cells. Using presence or absence of inhibition as a binary fixed effect did not impact the reported results or conclusions.

## Supporting information

S1 Fig Quantile–quantile plots for the genome-wide association analysis results for *Heliconius cydno alithea* male forewing color and mate choice. Both plots show Wald test *p*-values relative to the expected distribution. (**A**) Q–Q plot for the color GWA performed using GEMMA (genomic inflation factor =  1.006). (**B**) Q–Q plot for male choice GWA performed using GMMAT (genomic inflation factor =  0.924). Raw data and code used to generate these plots can be found in the Dryad repository dryad.z8w9ghxjz “gwas” directory.
(PNG)

S2 Fig Choice GWA results near other FDR <  0.01 peaks. Dark gray lines indicate FDR 0.01, light gray lines FDR 0.05. Gene models are shown along the *x*-axis, with exons as vertical boxes and gene spans indicated as lines. Genes on the plus strand are shown over genes on the minus strand. (**A**) Top choice variants on chromosome 7 fall within the second intron of *diacyl glycerol kinase* (*DAGK*). (**B**) Top choice variants on chromosome 9 fall within the second intron of *spineless*. (**C**) Top choice variants on chromosome 11 fall 2.5 kb upstream of *UBE2M*. *COL11A1*: collagen 11A1, *Daxx*: death domain associated protein. Raw data and code used to generate these plots can be found in the Dryad repository dryad.z8w9ghxjz “gwas” directory.
(PNG)

S3 Fig Linkage disequilibrium (LD) across the *K* locus. *D*′ and *r*^*2*^ were calculated in 5 kb non-overlapping windows. Pairwise LD values for all variants in the *K* locus were calculated using PLINK 1.9 (*--r2 inter-chr gz dprime --ld-window-r2 0.0*) and among the 113 sequenced *Heliconius cydno alithea* samples used in GWA. Scaffolds (black bars) and gene models (gray boxes) are shown along the *x*-axis, with (left to right) *al-1*, *al-2*, and *sens-2* filled with gold. Genome-wide averages of *D*′ and *r*^2^ in 5 kb windows are shown as dotted lines. Raw data and code used to generate these plots can be found in the Dryad repository dryad.z8w9ghxjz “gwas” directory.
(PNG)

S4 Fig The proportion of bases classified as repeats by RepeatMasker genome-wide (A) and in the *K* locus (B).
(**A**) The proportion of masked sequence in 50 kb sliding (5 kb step) windows. Red lines represent loess fits per-chromosome. (**B**) The proportion of masked sequence in 10 kb sliding (1 kb step) windows in the *K* locus. Gene models are shown as gray boxes along the *x*-axis; *al-1* and *sens-2* are highlighted in blue and gold, respectively. These results shown did not change when we limit to just putative TEs. Raw data and code used to generate these plots can be found in the Dryad repository dryad.z8w9ghxjz “gwas” directory.
(PNG)

S5 Fig Expression patterns of additional key genes in the *K* locus. *Aristaless-1* controls white versus yellow forewing color. *Senseless-2* is the nearest gene to KP3. *tFIIB* is a general transcription factor near KP1 that is differentially expressed in the developing retina and larval/P0 heads. *Senseless*-2 is a zinc finger transcription factor near to KP3. While a previous analysis showed that the gene *senseless-2* was differentially expressed between developing heads of white and yellow butterflies based on qPCR (VanKuren et al., 2022, 10.1101/2022.04.25.489404), we did not find *sens-2* to be DE based on these RNA-seq data. Raw data and code used to generate these plots can be found in the Dryad repository dryad.z8w9ghxjz “rnaseq” directory.
(PNG)

S6 Fig Gene networks underlying *Heliconius cydno alithea* mate preference variation. (**A**) DEG enrichment in co-expressed gene modules. We constructed a gene co-expression network (GCN) using WGCNA and all *H. c. alithea* RNA-seq data, clustered co-expressed gene modules based on module eigengene vectors, then tested if modules were enriched with DEGs using Fisher Exact Tests. (**B**) Replicate (point) and median (line) expression profiles and GO enrichment are shown for the three most significantly enriched modules. Note that module 1 showed no GO enrichment, likely due its small size. sDEGs: stage-specific differentially expressed genes; tDEGs: genes with significantly different expression profiles between yellow and white *H. c. alithea* males. Raw data and code used to generate these plots can be found in the Dryad repository dryad.z8w9ghxjz “rnaseq” directory.
(PNG)

S7 Fig Photoreceptor spectral tuning. (**A**) (top) Spectral sensitivity of blue sensitive photoreceptors, averaged across all recorded cells. Shading shows standard error. (Bottom) Wavelength of peak sensitivity was estimated for each cell by fitting the response with a template tuning curve (*n* =  21, 30, 22, 21, 5, 15, 12). (**B**) Same as panel A for green sensitive photoreceptors (*n* =  27, 25, 22, 7, 11, 29, 28). (**C**) Spectral sensitivity for a second type of LW sensitive photoreceptor, similar to those seen in other *Heliconius* species and presumably derived from a combination of the LW opsin and a red screening pigment (*n* =  2, 2, 5, 0, 1, 14, 9). (**D**) Spectral sensitivity for broadband sensitive photoreceptors, likely derived from co-expression of the blue and LW opsin. Tuning curves are separated into cells with (yellow, *n* = 7) and without (black, *n* = 13) evidence of long wavelength inhibition. Note that at the longest three wavelengths used for these recordings (>640 nm), the monochromator produced secondary peaks of excitation in the UV part of the spectrum. Raw data used to generate these plots can be found in the Dryad repository dryad.z8w9ghxjz “electrophysiology” directory.
(PNG)

S8 Fig Spectral tuning of single photoreceptors. (**A**) Each panel shows the distribution of the peak of the spectral tuning for UV photoreceptors (*λ*_Max_) for each group of butterflies, binned in 10 nm increments. Dotted lines indicate the expected tuning of UV1 and UV2 opsins. (**B**) Distribution of *λ*_Max_ for blue and green sensitive photoreceptors. Cells from all seven groups of butterfly we examined are combined for each panel. Raw data used to generate these plots can be found in the Dryad repository dryad.z8w9ghxjz “electrophysiology” directory.
(PNG)

S9 Fig Responses across intensities. (**A**) Solid lines show the response of each photoreceptor at *λ*_Max._ across 3 log units of intensity. For photoreceptors with evidence of inhibition, we also recorded responses at an inhibitory wavelength. Note that they are plotted as the absolute value. Error bars show SEM. (**B**) Response latency measurements for the cells in panel A. Small responses limited these measurements to only 2 log units of intensity. Inhibitory latencies were significantly different from *λ*_Max_ for all intensity levels (*t*-tests, *p* <  0.01). Raw data used to generate these plots can be found in the Dryad repository dryad.z8w9ghxjz “electrophysiology” directory.
(PNG)

S10 Fig Photoreceptor temporal responses. (**A**) Example responses from single trials show the differences in the temporal responses of different photoreceptor types. Times are aligned for 0 ms to be the onset of each response. (**B**) The temporal response was measured as the amount of time the response to *λ*_Max_ remained above 50% of the maximum. Width was measured for UV photoreceptors both before and after turning on the background LED. Letters above indicate groups significantly different from each other (*F*_6,328_ =  29.92, *p* <  0.001, Tukey’s HSD). Raw data used to generate these plots can be found in the Dryad repository dryad.z8w9ghxjz “electrophysiology” directory.
(PNG)

S11 Fig *F*_*ST*_ between yellow and white *Heliconius cydno alithea* genome-wide and in the *K* locus. (**A**) Genome-wide *F*_*ST*_ calculated in 10 kb sliding windows (2 kb step). The peak on chromosome 1 corresponds to the *K* locus. (**B**) *F*_*ST*_ around the *K* locus (chr1:14500000-16500000) calculated in 10 kb sliding windows (2 kb step). Gene models are shown along the *x* axis, with genes on the positive strand above those on the negative strand; *herzog*, *alsin-2*, and *tFIIB* are highlighted in red (see Fig 2). *F*_*ST*_ was calculated 57 yellow and 56 white-winged males using VCFtools 0.1.16 and the final variant callset used for the GWA analyses (8.7M LD-pruned variants) for both plots. Raw data and code used to generate these plots can be found in the Dryad repository dryad.z8w9ghxjz “gwas” directory.
(PNG)

S12 Fig Emission spectra of the monochromator. Each panel shows the emission spectrum for one of the monochromatic stimuli used in the electrophysiology experiments. During experiments, isoquantal intensities were achieved using a variable neutral density filter. Raw data used to generate these plots can be found in the Dryad repository dryad.z8w9ghxjz “electrophysiology” directory.
(PNG)

S1 Table Choice data for 113 males included in the mate choice genome-wide association analysis. Each row indicates one courtship event and whether the male chose the white female (Choice =  1) or yellow female (Choice =  0). The “Contrast YT” variable indicates whether the yellow female in that particular trial (e.g., 2-Oct-08.1) had the melanic Ac patch (see [16] for detail). The “Male Color” variable indicates whether the male had a yellow (1) or white (0) forewing. The “Court Number” variable is a rolling sum of the number of choices that particular male made. Raw whole genome sequencing data for each of the 113 individuals can be found in NCBI BioProject PRJNA802836.
(XLSX)

S2 Table Genes in the *K* locus (Fig 2B). (XLSX)

S3 Table RNA-seq data information. hcaw, hcay, hcyg: white Heliconius cydno alithea, yellow H. c. alithea, Heliconius cydno galanthus, respectively. Stage: p0: 12–24 h after pupation; p2: 48–60 hap; p4: 96–108 hap; p6: 144–156 hap; and p7: 168–180 hap; ad: 24–48 h after emergence. Outlier: samples considered as outliers (1) or not (0) for the final analyses presented in the publication. See methods. Raw sequencing reads can be downloaded from NCBI BioProject **PRJNA1019262.**
(XLSX)

S4 Table Differentially expressed genes between yellow and white *Heliconius cydno alithea* male purple crawler (pc) and 12-24h pupal (p0) heads. Global FDR (gFDR) was calculated using all *p*-values from all DESeq2 analyses for all tissues using the Benjamini–Hochberg method. The top blastp hits to the *Drosophila melanogaster* r6.44 proteome are shown.
(XLSX)

S5 Table Differentially expressed genes between yellow and white *Heliconius cydno alithea* male brains. Global FDR (gFDR) values for DESeq2 were calculated using all *p*-values from all DESeq2 analyses for all tissues using the Benjamini–Hochberg method. gFDRs are shown for each stage-specific comparison. maSigPro model fit *p*-values and *R*-squared values are shown. The top blastp hits to the *Drosophila melanogaster* r6.44 proteome are shown.
(XLSX)

S6 Table Differentially expressed genes between yellow and white *Heliconius cydno alithea* male optic lobes. Global FDR (gFDR) values for DESeq2 were calculated using all *p*-values from all DESeq2 analyses for all tissues using the Benjamini–Hochberg method. gFDRs are shown for each stage-specific comparison. maSigPro model fit *p*-values and *R*-squared values are shown. The top blastp hits to the *Drosophila melanogaster* r6.44 proteome are shown.
(XLSX)

S7 Table Differentially expressed genes between yellow and white *Heliconius cydno alithea* male retinas. Global FDR (gFDR) values for DESeq2 were calculated using all *p*-values from all DESeq2 analyses for all tissues using the Benjamini–Hochberg method. gFDRs are shown for each stage-specific comparison. maSigPro model fit *p*-values and *R*-squared values are shown. The top blastp hits to the *Drosophila melanogaster* r6.44 proteome are shown.
(XLSX)

S8 Table Evidence weights used in EVM-based annotation of the *Heliconius cydno alithea* genome. (XLSX)

## Abbreviations:

DE: differentially expressed
FDR: false discovery rate
GLME: generalized linear mixed-effects
GO: gene ontology
GWA: genome-wide association
LD: linkage disequilibrium
ONT: Oxford Nanopore Technologies
PR: photoreceptor
SNP: single-nucleotide polymorphism
UV: ultraviolet
