# Supplementary figures and images for: Genetic, developmental, and neural changes underlying the evolution of butterfly mate preference
Source: PLoS Biol. 2025 Mar 11;23(3):e3002989. doi: 10.1371/journal.pbio.3002989 (PMC12136153; doi:10.1371/journal.pbio.3002989)

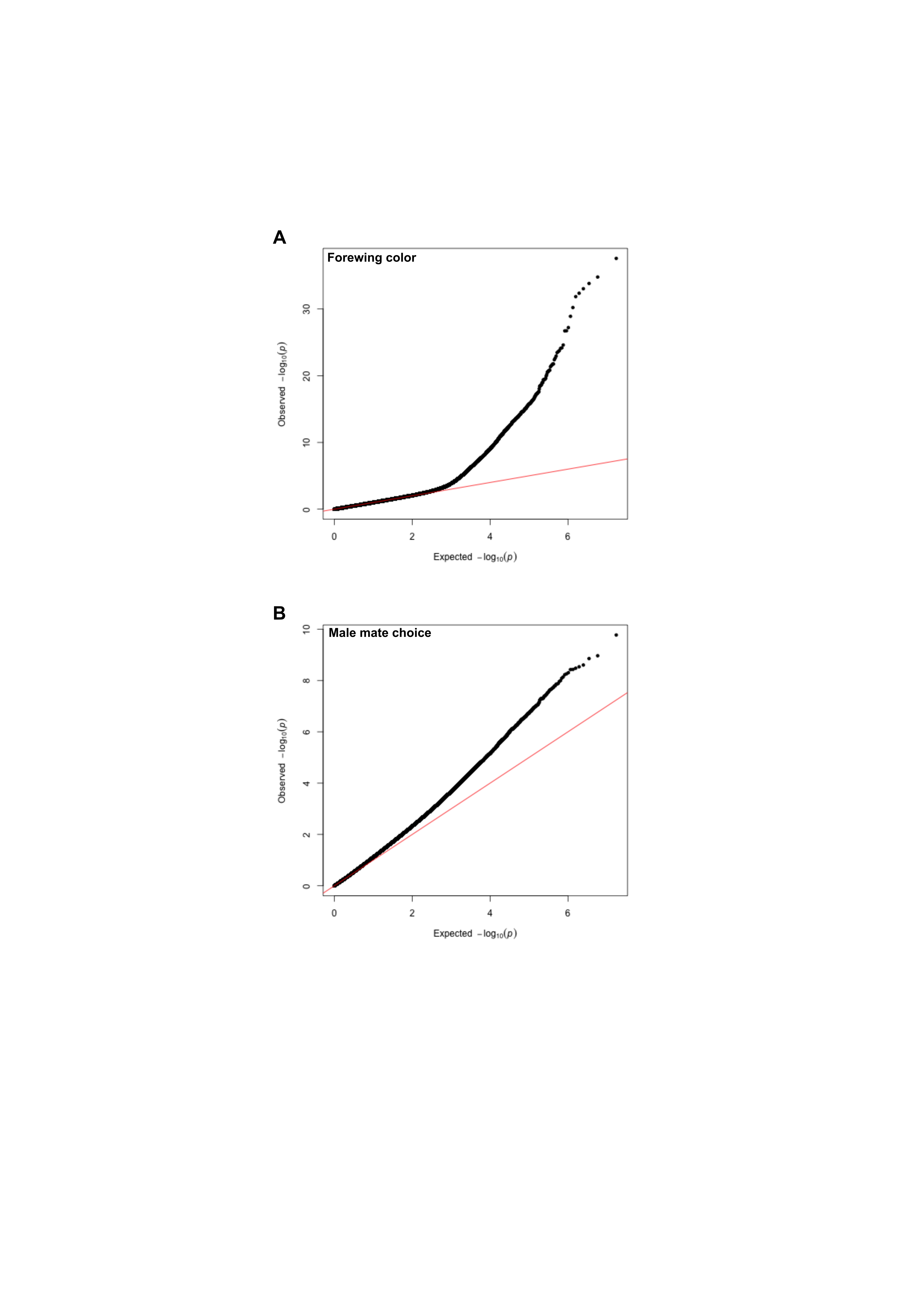

Supplement: S1 Fig — Both plots show Wald test p-values relative to the expected distribution. (A) Q–Q plot for the color GWA performed using GEMMA (genomic inflation factor = 1.006). (B) Q–Q plot for male choice GWA performed using GMMAT (genomic inflation factor = 0.924). Raw data and code used to generate these plots can be found in the Dryad repository dryad.z8w9ghxjz “gwas” directory. (PNG) [file pbio.3002989.s001.png]

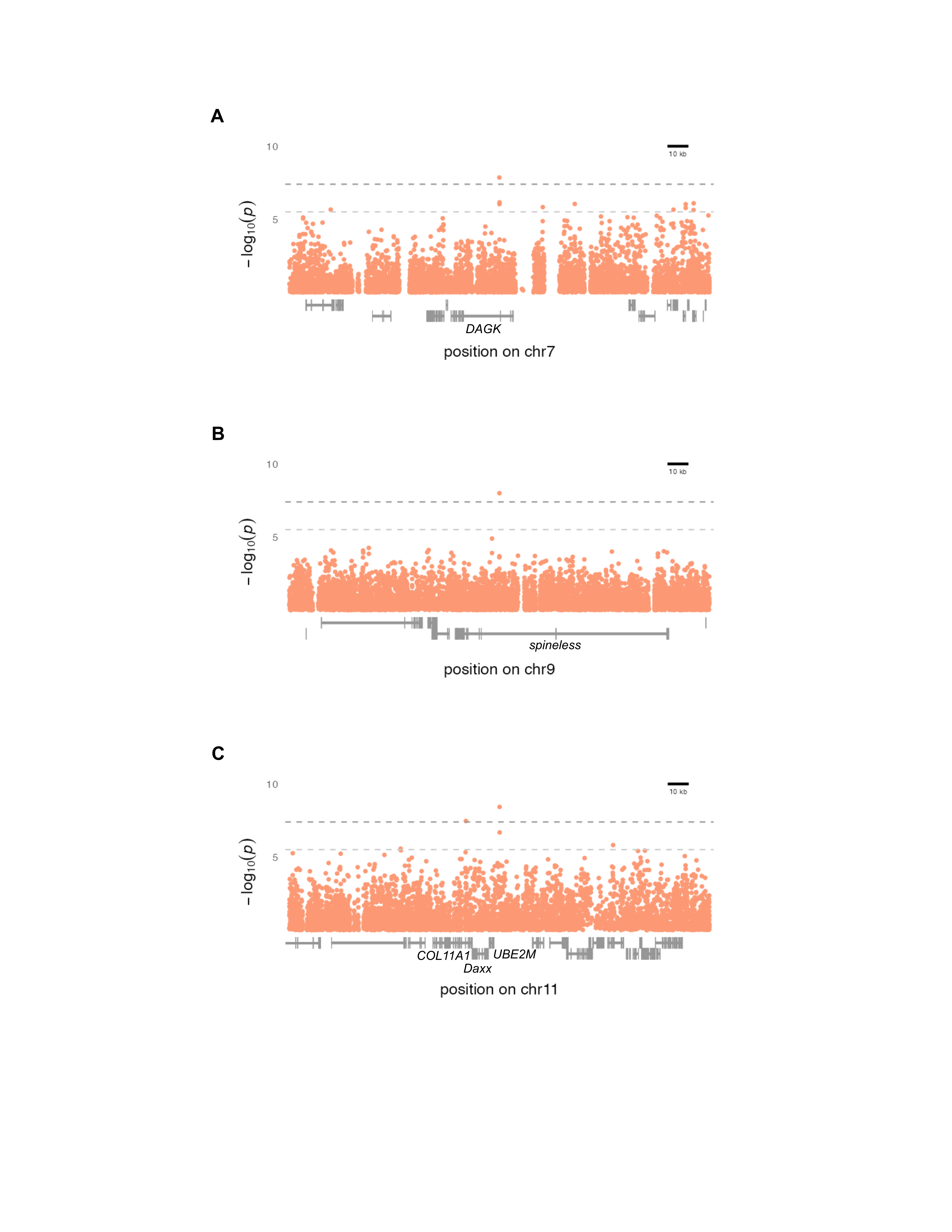

Supplement: S2 Fig — Dark gray lines indicate FDR 0.01, light gray lines FDR 0.05. Gene models are shown along the x-axis, with exons as vertical boxes and gene spans indicated as lines. Genes on the plus strand are shown over genes on the minus strand. (A) Top choice variants on chromosome 7 fall within the second intron of diacyl glycerol kinase (DAGK). (B) Top choice variants on chromosome 9 fall within the second intron of spineless. (C) Top choice variants on chromosome 11 fall 2.5 kb upstream of UBE2M. COL11A1: collagen 11A1, Daxx: death domain associated protein. Raw data and code used to generate these plots can be found in the Dryad repository dryad.z8w9ghxjz “gwas” directory. (PNG) [file pbio.3002989.s002.png]

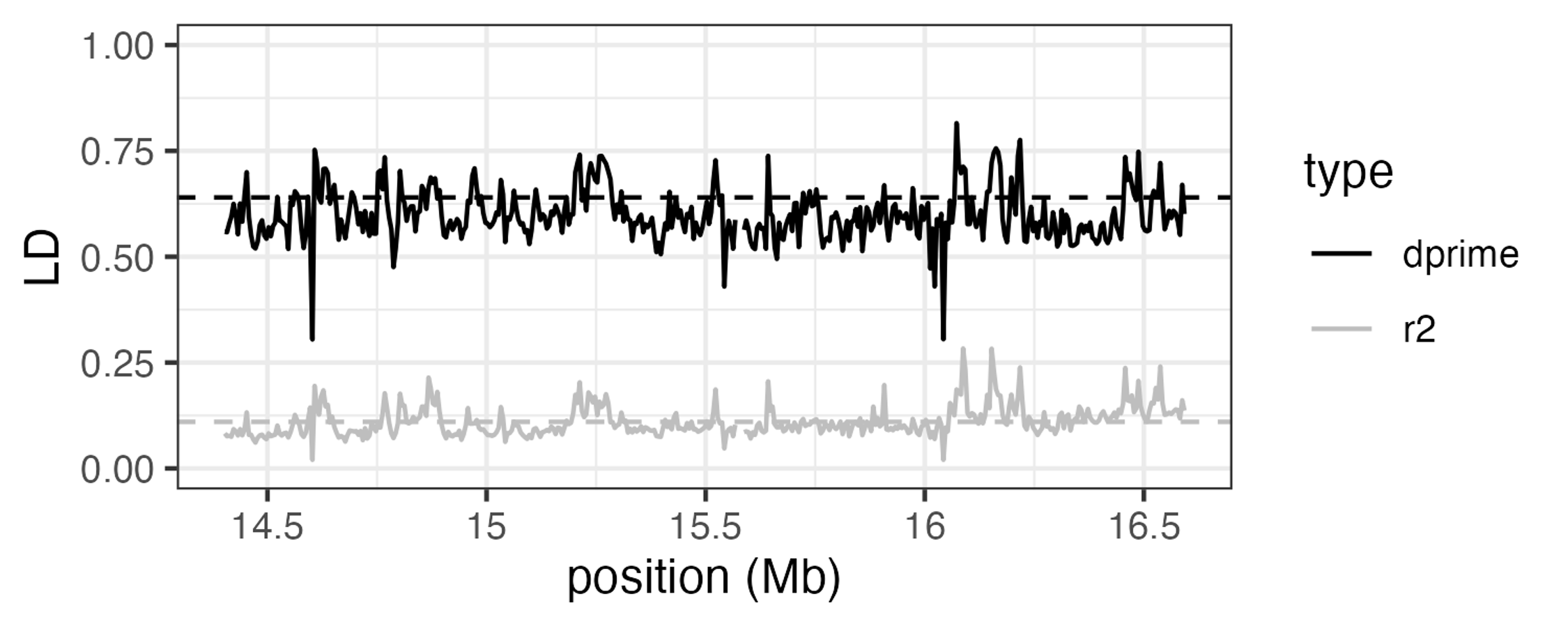

Supplement: S3 Fig — D′ and r2 were calculated in 5 kb non-overlapping windows. Pairwise LD values for all variants in the K locus were calculated using PLINK 1.9 (--r2 inter-chr gz dprime --ld-window-r2 0.0) and among the 113 sequenced Heliconius cydno alithea samples used in GWA. Scaffolds (black bars) and gene models (gray boxes) are shown along the x-axis, with (left to right) al-1, al-2, and sens-2 filled with gold. Genome-wide averages of D′ and r2 in 5 kb windows are shown as dotted lines. Raw data and code used to generate these plots can be found in the Dryad repository dryad.z8w9ghxjz “gwas” directory. (PNG) [file pbio.3002989.s003.png]

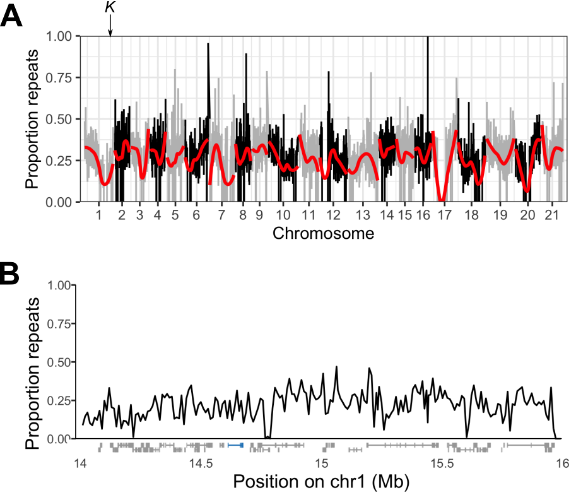

Supplement: S4 Fig — (A) The proportion of masked sequence in 50 kb sliding (5 kb step) windows. Red lines represent loess fits per-chromosome. (B) The proportion of masked sequence in 10 kb sliding (1 kb step) windows in the K locus. Gene models are shown as gray boxes along the x-axis; al-1 and sens-2 are highlighted in blue and gold, respectively. These results shown did not change when we limit to just putative TEs. Raw data and code used to generate these plots can be found in the Dryad repository dryad.z8w9ghxjz “gwas” directory. (PNG) [file pbio.3002989.s004.png]

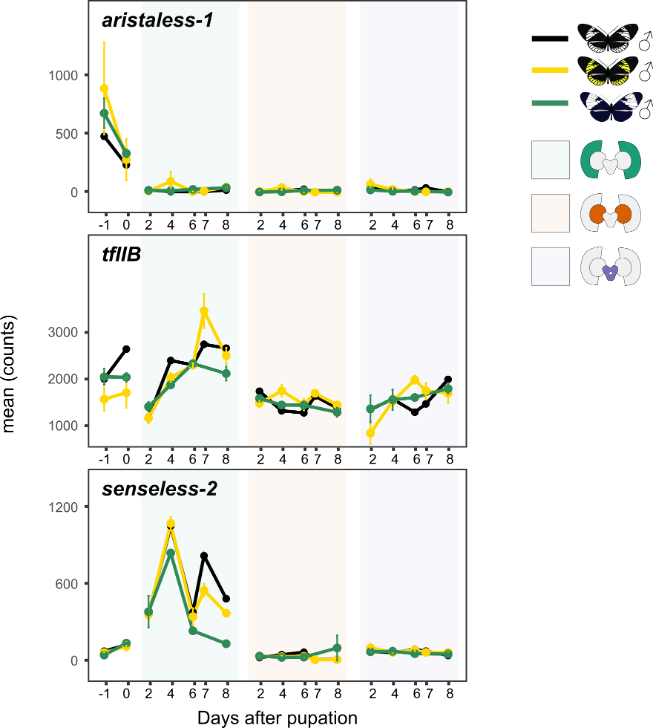

Supplement: S5 Fig — Aristaless-1 controls white versus yellow forewing color. Senseless-2 is the nearest gene to KP3. tFIIB is a general transcription factor near KP1 that is differentially expressed in the developing retina and larval/P0 heads. Senseless-2 is a zinc finger transcription factor near to KP3. While a previous analysis showed that the gene senseless-2 was differentially expressed between developing heads of white and yellow butterflies based on qPCR (VanKuren et al., 2022, 10.1101/2022.04.25.489404), we did not find sens-2 to be DE based on these RNA-seq data. Raw data and code used to generate these plots can be found in the Dryad repository dryad.z8w9ghxjz “rnaseq” directory. (PNG) [file pbio.3002989.s005.png]

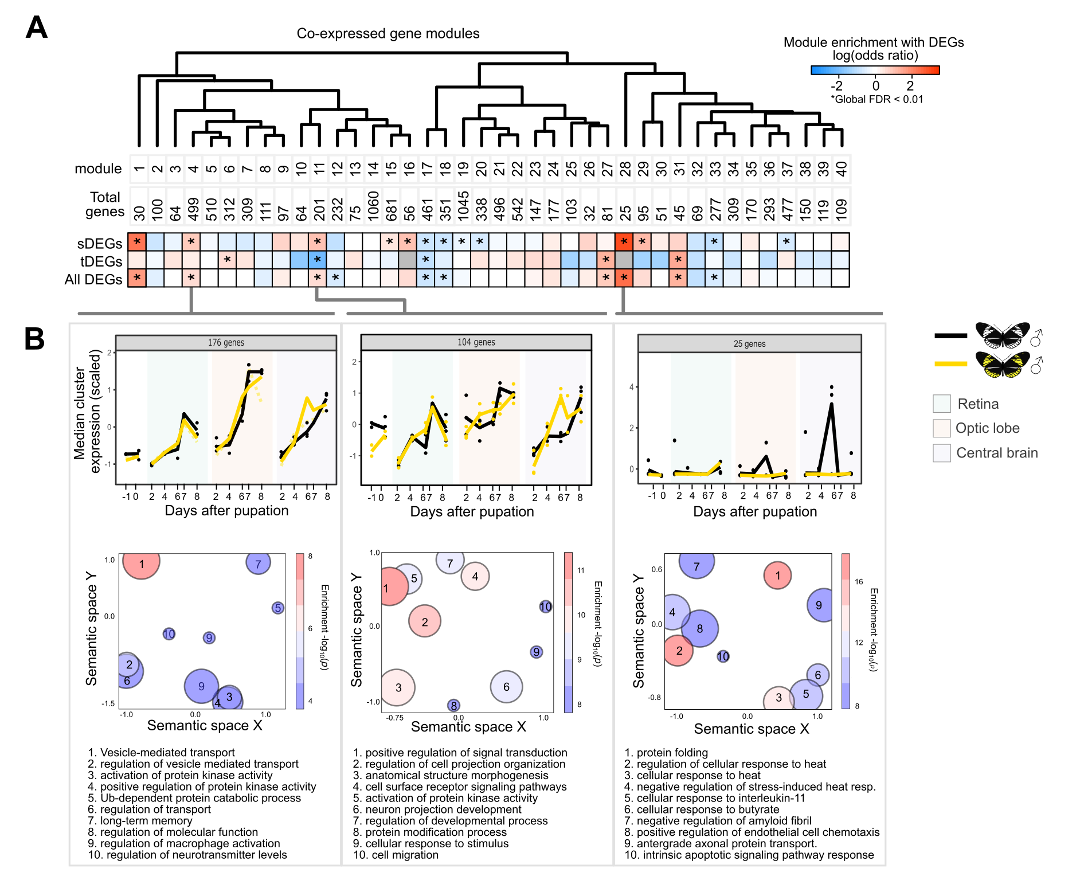

Supplement: S6 Fig — (A) DEG enrichment in co-expressed gene modules. We constructed a gene co-expression network (GCN) using WGCNA and all H. c. alithea RNA-seq data, clustered co-expressed gene modules based on module eigengene vectors, then tested if modules were enriched with DEGs using Fisher Exact Tests. (B) Replicate (point) and median (line) expression profiles and GO enrichment are shown for the three most significantly enriched modules. Note that module 1 showed no GO enrichment, likely due its small size. sDEGs: stage-specific differentially expressed genes; tDEGs: genes with significantly different expression profiles between yellow and white H. c. alithea males. Raw data and code used to generate these plots can be found in the Dryad repository dryad.z8w9ghxjz “rnaseq” directory. (PNG) [file pbio.3002989.s006.png]

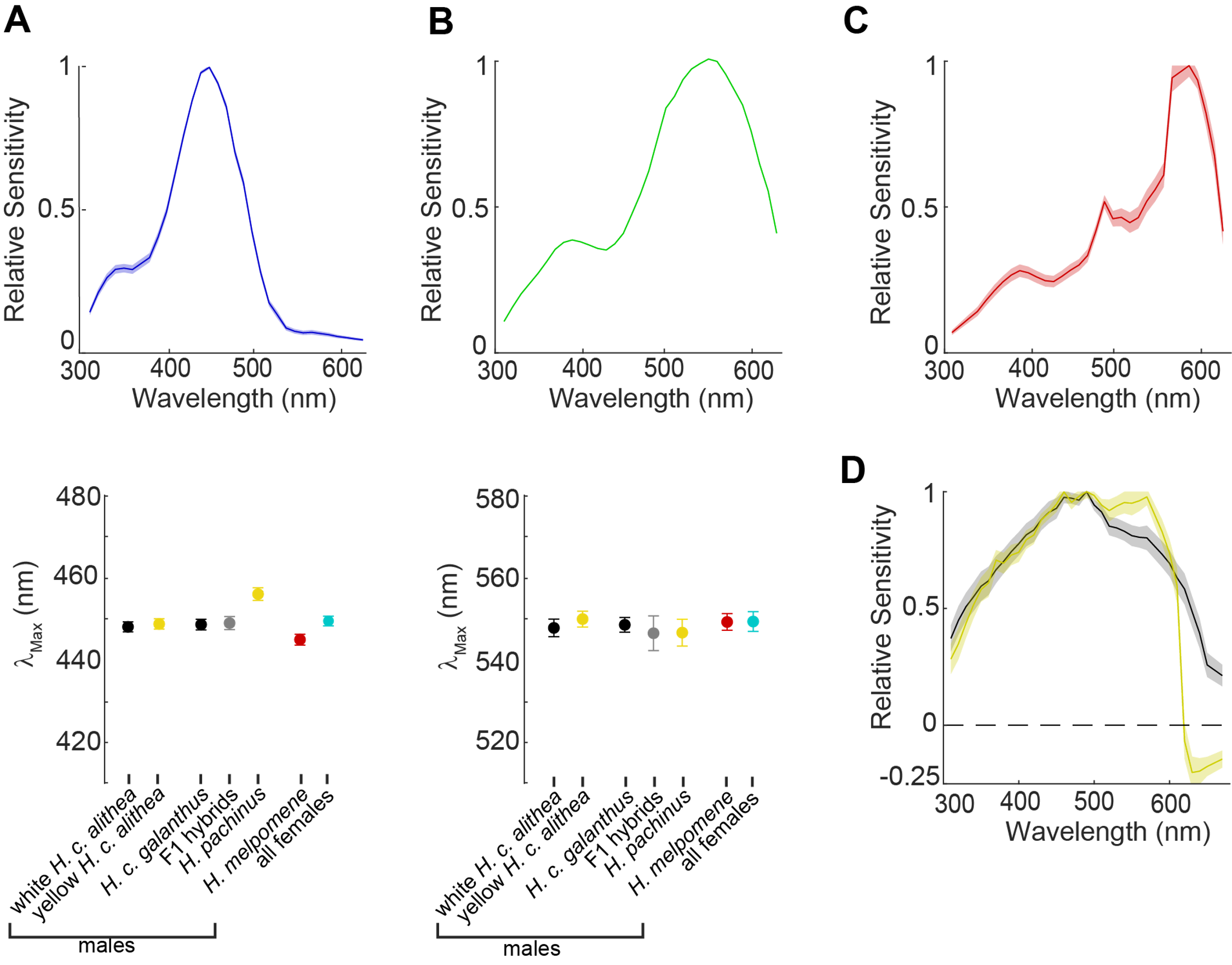

Supplement: S7 Fig — (A) (top) Spectral sensitivity of blue sensitive photoreceptors, averaged across all recorded cells. Shading shows standard error. (Bottom) Wavelength of peak sensitivity was estimated for each cell by fitting the response with a template tuning curve (n = 21, 30, 22, 21, 5, 15, 12). (B) Same as panel A for green sensitive photoreceptors (n = 27, 25, 22, 7, 11, 29, 28). (C) Spectral sensitivity for a second type of LW sensitive photoreceptor, similar to those seen in other Heliconius species and presumably derived from a combination of the LW opsin and a red screening pigment (n = 2, 2, 5, 0, 1, 14, 9). (D) Spectral sensitivity for broadband sensitive photoreceptors, likely derived from co-expression of the blue and LW opsin. Tuning curves are separated into cells with (yellow, n = 7) and without (black, n = 13) evidence of long wavelength inhibition. Note that at the longest three wavelengths used for these recordings (>640 nm), the monochromator produced secondary peaks of excitation in the UV part of the spectrum. Raw data used to generate these plots can be found in the Dryad repository dryad.z8w9ghxjz “electrophysiology” directory. (PNG) [file pbio.3002989.s007.png]

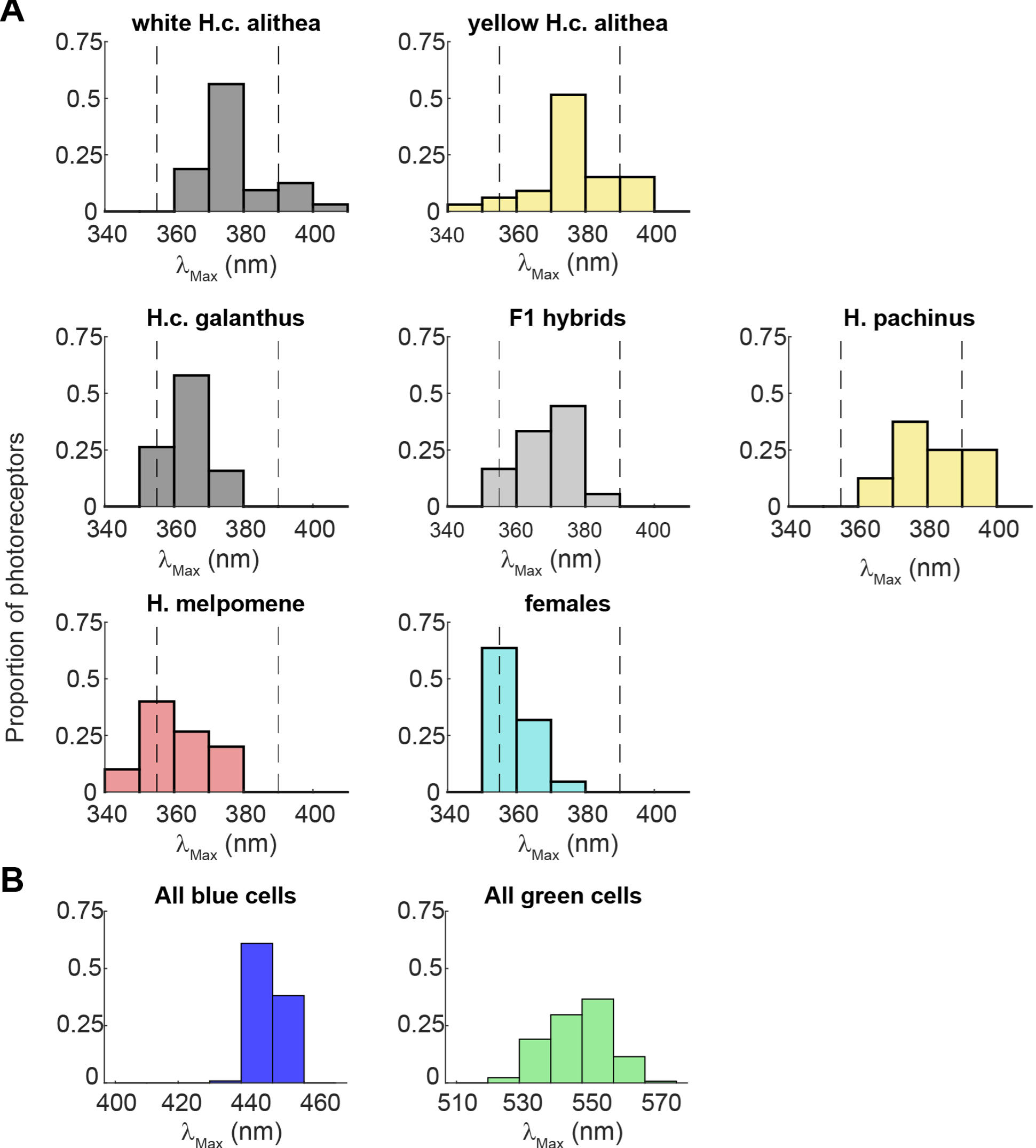

Supplement: S8 Fig — (A) Each panel shows the distribution of the peak of the spectral tuning for UV photoreceptors (λMax) for each group of butterflies, binned in 10 nm increments. Dotted lines indicate the expected tuning of UV1 and UV2 opsins. (B) Distribution of λMax for blue and green sensitive photoreceptors. Cells from all seven groups of butterfly we examined are combined for each panel. Raw data used to generate these plots can be found in the Dryad repository dryad.z8w9ghxjz “electrophysiology” directory. (PNG) [file pbio.3002989.s008.png]

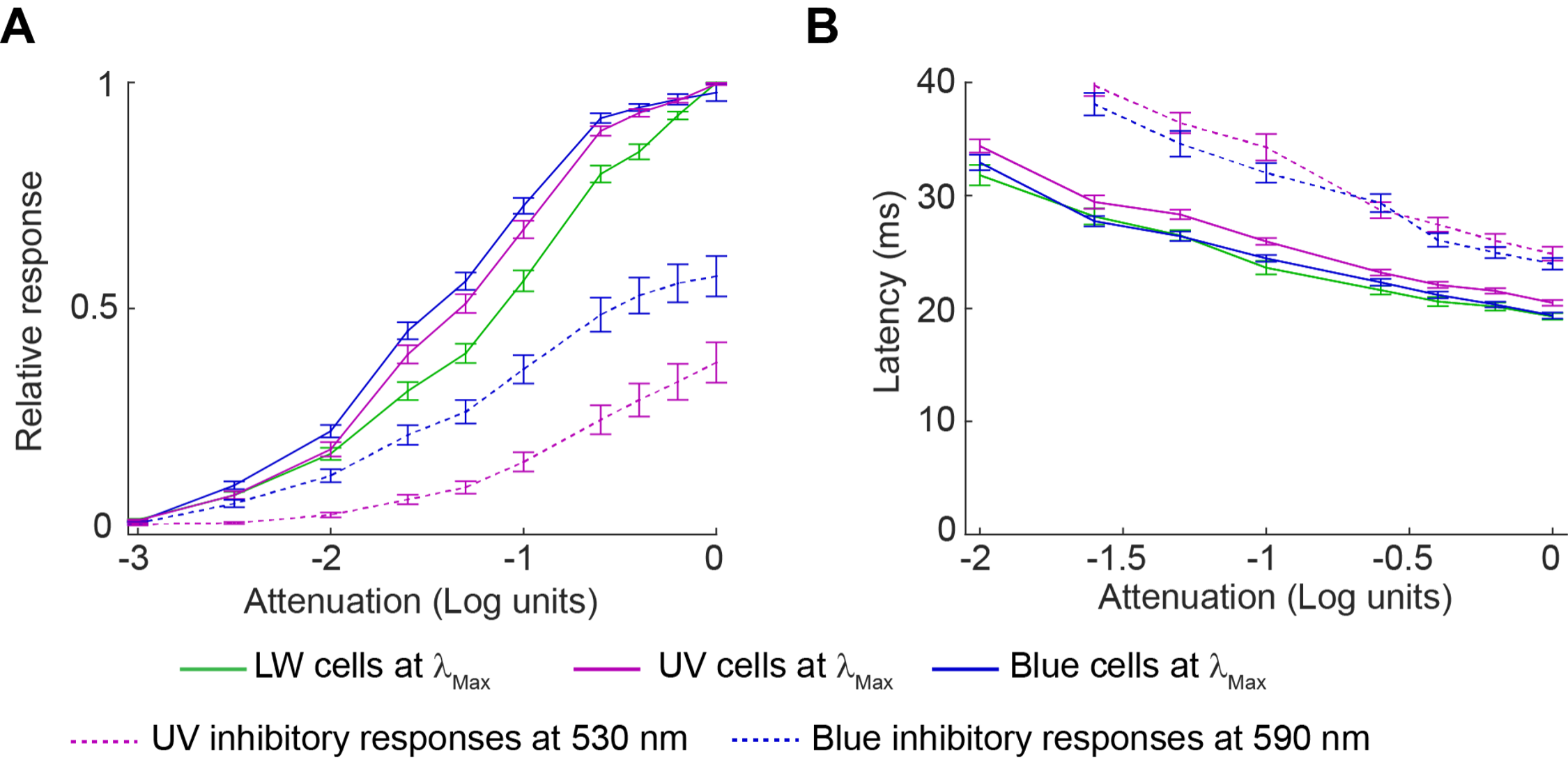

Supplement: S9 Fig — (A) Solid lines show the response of each photoreceptor at λMax. across 3 log units of intensity. For photoreceptors with evidence of inhibition, we also recorded responses at an inhibitory wavelength. Note that they are plotted as the absolute value. Error bars show SEM. (B) Response latency measurements for the cells in panel A. Small responses limited these measurements to only 2 log units of intensity. Inhibitory latencies were significantly different from λMax for all intensity levels (t-tests, p < 0.01). Raw data used to generate these plots can be found in the Dryad repository dryad.z8w9ghxjz “electrophysiology” directory. (PNG) [file pbio.3002989.s009.png]

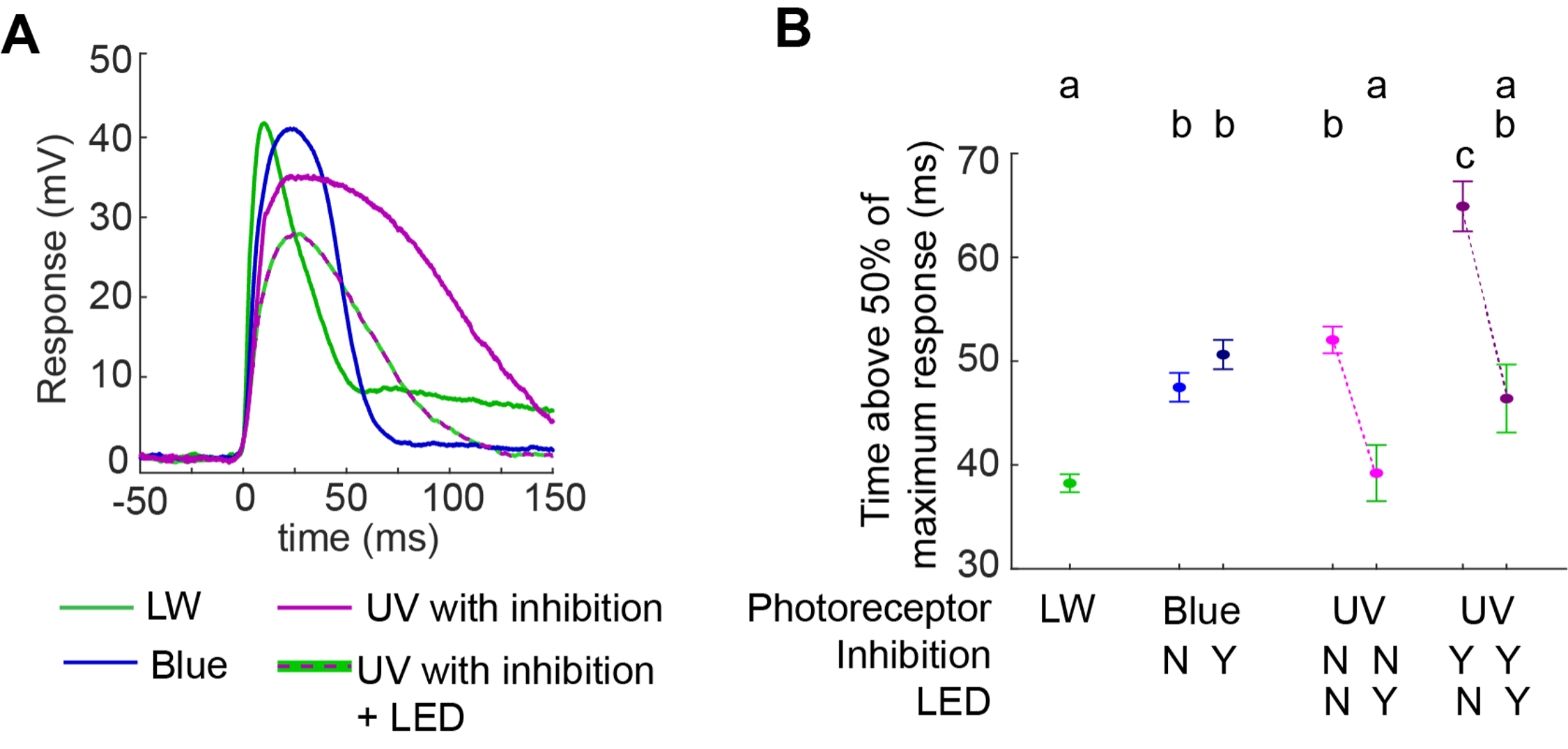

Supplement: S10 Fig — (A) Example responses from single trials show the differences in the temporal responses of different photoreceptor types. Times are aligned for 0 ms to be the onset of each response. (B) The temporal response was measured as the amount of time the response to λMax remained above 50% of the maximum. Width was measured for UV photoreceptors both before and after turning on the background LED. Letters above indicate groups significantly different from each other (F6,328 = 29.92, p < 0.001, Tukey’s HSD). Raw data used to generate these plots can be found in the Dryad repository dryad.z8w9ghxjz “electrophysiology” directory. (PNG) [file pbio.3002989.s010.png]

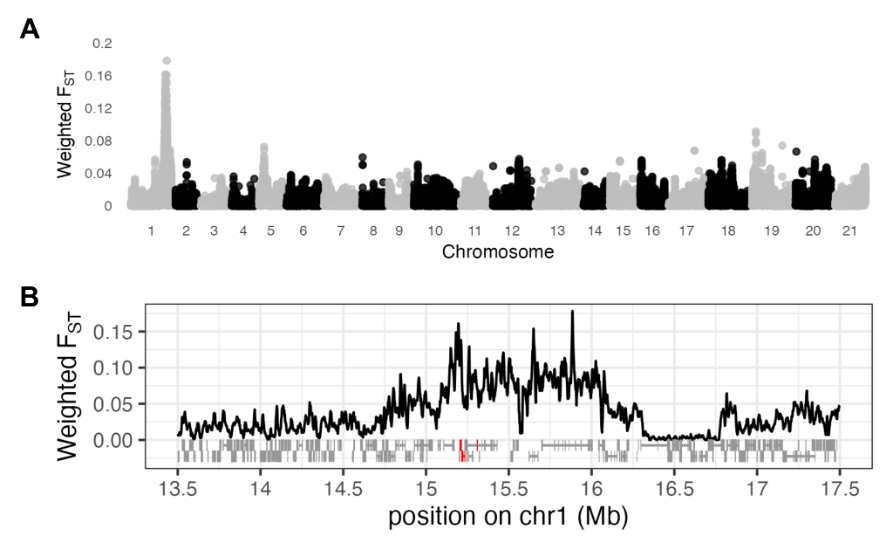

Supplement: S11 Fig — (A) Genome-wide FST calculated in 10 kb sliding windows (2 kb step). The peak on chromosome 1 corresponds to the K locus. (B) FST around the K locus (chr1:14500000-16500000) calculated in 10 kb sliding windows (2 kb step). Gene models are shown along the x axis, with genes on the positive strand above those on the negative strand; herzog, alsin-2, and tFIIB are highlighted in red (see Fig 2). FST was calculated 57 yellow and 56 white-winged males using VCFtools 0.1.16 and the final variant callset used for the GWA analyses (8.7M LD-pruned variants) for both plots. Raw data and code used to generate these plots can be found in the Dryad repository dryad.z8w9ghxjz “gwas” directory. (PNG) [file pbio.3002989.s011.png]

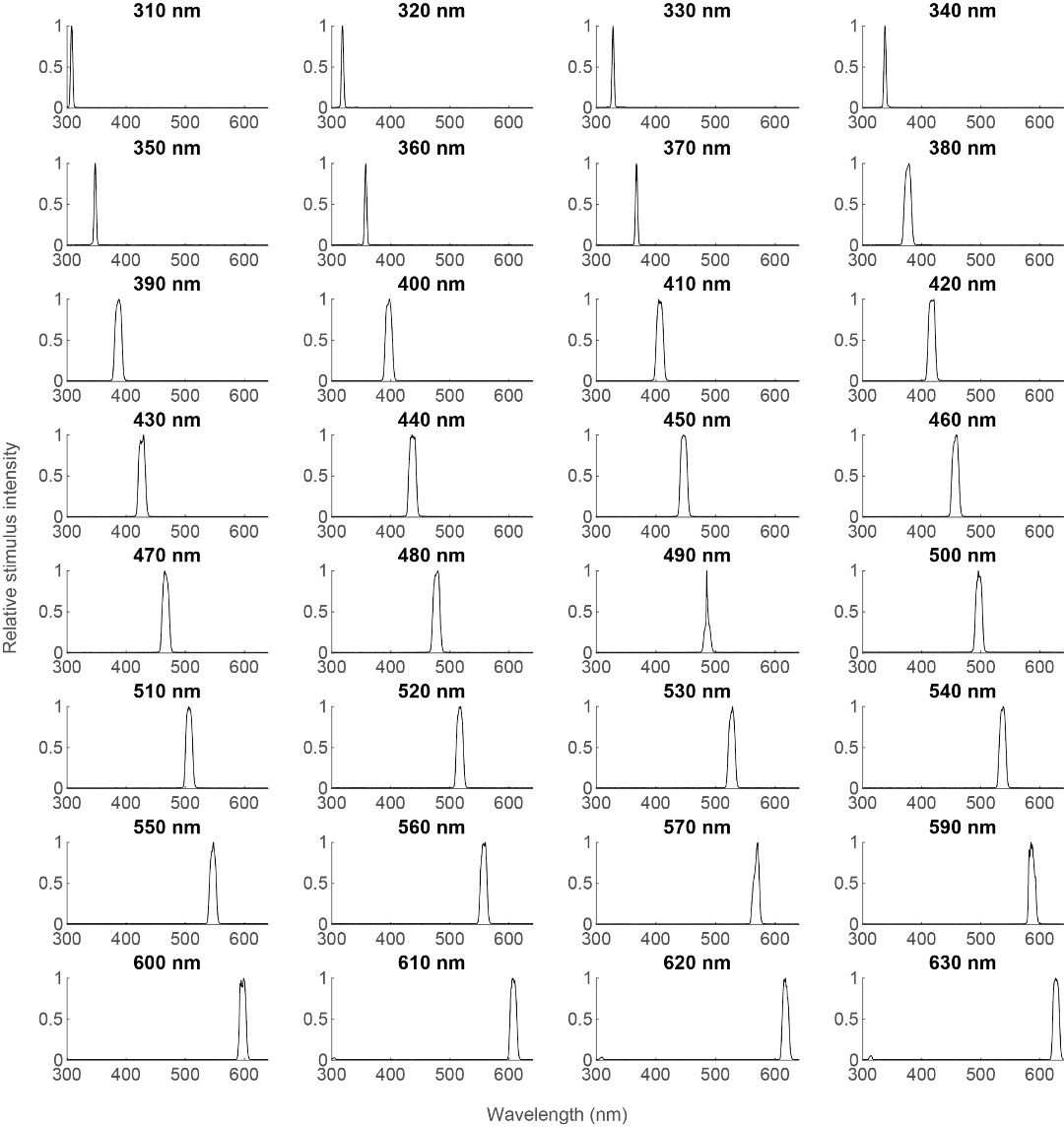

Supplement: S12 Fig — Each panel shows the emission spectrum for one of the monochromatic stimuli used in the electrophysiology experiments. During experiments, isoquantal intensities were achieved using a variable neutral density filter. Raw data used to generate these plots can be found in the Dryad repository dryad.z8w9ghxjz “electrophysiology” directory. (PNG) [file pbio.3002989.s012.png]
